# Comparison of the transcriptomic "stress response" evoked by antimycin A and oxygen deprivation in *saccharomyces cerevisiae*

**DOI:** 10.1186/1471-2164-9-627

**Published:** 2008-12-23

**Authors:** Liang-Chuan Lai, Matthew T Kissinger, Patricia V Burke, Kurt E Kwast

**Affiliations:** 1 Department of Physiology, National Taiwan University College of Medicine, Taipei, Taiwan (R.O.C.); 2 Coskata Inc., 4575 Weaver Pkwy, Suite 100, Warrenville, IL 60555, USA; 3 Department of Microbiology, University of Illinois, Urbana, IL 61801, USA; 4 Department of Molecular & Integrative Physiology, University ofIllinois, Urbana, IL 61801, USA

## Abstract

**Background:**

Acute changes in environmental parameters (e.g., O_2_, pH, UV, osmolarity, nutrients, etc.) evoke a common transcriptomic response in yeast referred to as the "environmental stress response" (ESR) or "common environmental response" (CER). Why such a diverse array of insults should elicit a common transcriptional response remains enigmatic. Previous functional analyses of the networks involved have found that, in addition to up-regulating those for mitigating the specific stressor, the majority appear to be involved in balancing energetic supply and demand and modulating progression through the cell cycle. Here we compared functional and regulatory aspects of the stress responses elicited by the acute inhibition of respiration with antimycin A and oxygen deprivation under catabolite non-repressed (galactose) conditions.

**Results:**

Gene network analyses of the transcriptomic responses revealed both treatments result in the transient (10 – 60 min) down-regulation of MBF- and SBF-regulated networks involved in the G1/S transition of the cell cycle as well as Fhl1 and PAC/RRPE-associated networks involved in energetically costly programs of ribosomal biogenesis and protein synthesis. Simultaneously, Msn2/4 networks involved in hexose import/dissimilation, reserve energy regulation, and autophagy were transiently up-regulated. Interestingly, when cells were treated with antimycin A well before experiencing anaerobiosis these networks subsequently failed to respond to oxygen deprivation. These results suggest the transient stress response is elicited by the acute inhibition of respiration and, we postulate, changes in cellular energetics and/or the instantaneous growth rate, not oxygen deprivation *per se*. After a considerable delay (≥ 1 generation) under anoxia, predictable changes in heme-regulated gene networks (e.g., Hap1, Hap2/3/4/5, Mot3, Rox1 and Upc2) were observed both in the presence and absence of antimycin A.

**Conclusion:**

This study not only differentiates between the gene networks that respond to respiratory inhibition and those that respond to oxygen deprivation but suggests the function of the ESR or CER is to balance energetic supply/demand and coordinate growth with the cell cycle, whether in response to perturbations that disrupt catabolic pathways or those that require rapidly up-regulating energetically costly programs for combating specific stressors.

## Background

Previous studies with *Saccharomyces cerevisiae *have found that a common transcriptomic program is initiated in response to a diverse array of environmental challenges. These include temperature shock, osmotic or oxidative stress, low pH, DNA damaging agents, nutrient starvation, a switch to lower quality carbon sources, and oxygen deprivation under catabolite non-repressed conditions [1-6]. This program has been called the "environmental stress response" (ESR) [2] or "common environmental response" (CER) [4], but the factors responsible for initiating the response and the function of its various components remain unclear. Our previous gene network analyses of an ESR/CER-like response elicited by anaerobiosis [5,6] suggest it is involved in rebalancing energy supply and demand in response to an abrupt change in cellular energy status while concomitantly coordinating growth rate and entry into the cell cycle. Although not measured in most studies, acute yet transient changes in the cellular energetics would be expected to occur in response to any perturbations that disrupt catabolic pathways for producing energy (e.g., anaerobiosis, glucose starvation, or a switch to a lower quality energy source) or when survival depends on rapidly up-regulating energetically costly programs for combating specific stressors (e.g., temperature shock, osmotic or oxidative stress, DNA damaging agents, etc.). Thus, a common metabolic consequence that would be predicted to result from any of these environmental challenges is an abrupt, although transitory, decrease in energy that would require compensatory actions for survival.

Further support comes from the fact that the acute withdrawal of oxygen does not elicit such a response under catabolite-repressed conditions (glucose) but does under non-repressed conditions (galactose) [5,6]. A fundamental difference in the metabolism of these sugars that may explain this difference is the fact that oxygen deprivation does not substantially affect the overall yield or rate of ATP formation from glucose dissimilation but does severely limit both during galactose dissimilation. Thus, when cells are grown in glucose the aerobic-to-anaerobic transition proceeds without a change in growth rate [5,6], but in galactose the rapid withdrawal of oxygen results in the abrupt decrease in energy production and slowing of growth to a rate that is nearly half of that supported by mixed respiro-fermentative metabolism [5,6]. Although several recent studies have suggested that the ESR is evoked by growth rate differences [7], if this were true it is difficult to reconcile the fact these networks are not differentially expressed between the much faster respiro-fermentative growth phase in galactose and the slower fermentative growth phase but rather only transiently respond during the change [8,9]. An alternative explanation is that it is the abrupt decrease in cellular energy stores that elicits the ESR/CER under these conditions, not the withdrawal of oxygen. In other words, that it is the abrupt inhibition of oxidative phosphorylation and associated changes in cellular energetics that triggers this ESR/CER-like response, not changes in growth rate *per se*.

Further support comes from functional and gene network analyses of the anaerobic response in galactose [5,6]. Using a novel clustering approach for recovering active gene networks, as well as knockouts of selected transcription factors [5], we revealed the ESR/CER-like response is comprised of the transient up-regulation of Msn2/4-regulated networks involved in both hexose import/dissimilation and reserve energy regulation as well as the transient down-regulation of Fhl1 and PAC/RRPE-associated networks involved in energetically costly programs for ribosomal biogenesis and protein synthesis. In addition, MBF- and SBF-regulated networks involved in the G1/S transition of the cell cycle are also transiently down-regulated, resulting in a delay of its progression as mass (e.g., glycogen and trehalose stores) and energy are assessed before committing to another round of the cycle. Given their function, these results suggest that transient changes in the activity of these gene networks are required for balancing energy supply and demand and coordinating growth with progression through the cell cycle during the abrupt switch to strictly fermentative growth. Additional support comes from a study that found that treatment with the cytochrome *bc*_1 _inhibitor myxothiazol under normoxia in galactose grown cells also elicits a transient response in many of the genes that comprise the ESR/CER [10].

To further investigate the regulatory factors involved and distinguish between networks that respond to oxygen deprivation and those that respond to the abrupt cessation of respiration, we poisoned the respiratory chain with the cytochrome *bc*_1 _inhibitor antimycin A and conducted a temporal analysis of the transcriptomic response over three generations of aerobiosis followed by three generations of anaerobiosis. This treatment served several purposes. First, poisoning respiration under normoxic conditions mimics the loss of respiratory-dependent energy production that occurs during the shift to anaerobiosis, independent of a change in oxygen availability. Given that changes in the activity of more chronically responding gene networks are observed within three generations of anaerobic growth [6], assaying gene expression over this period should allow us to differentiate between networks that respond to the cessation of respiration and any that may respond to the chronic loss of respiratory capacity, independent of a change in oxygen availability. Moreover, by then shifting the respiratory incompetent cells to anaerobiosis and assaying gene expression over three additional generations of growth, we should be able to distinguish between any networks that acutely respond to the loss of oxygen and those that more chronically respond to oxygen-dependent changes in cellular heme concentrations, for example those that are regulated by Hap1, Hap2/3/4/5, Rox1, Upc2 and Mot3 [6]. Overall, we expected and found that the same ESR/CER gene networks respond to poisoning of the respiratory chain under normoxia as to the shift to anoxia in the absence of inhibitor, networks that appear to be involved in balancing energy supply and demand, growth rate, and progression through the cell cycle.

## Results

### Statistical comparisons of the transcriptomic responses

To compare the dynamics of the transcriptomic responses elicited by antimycin A treatment and anaerobiosis [6], we harvested samples after the same number of generations following each treatment as previously we had shown such a growth-rate dependent alignment is superior to time-dependent alignments ([5] see also [7]). Separate ANOVAs were conducted to identify genes whose transcript levels responded significantly (*P *≤ 0.01 after step-down Bonferroni adjustment) to each treatment. In total, 1754 genes were found to respond significantly at one or more time points to anaerobiosis, 901 to antimycin A treatment in air, and 669 to anaerobiosis in the presence of antimycin A.

**Figure 1:**
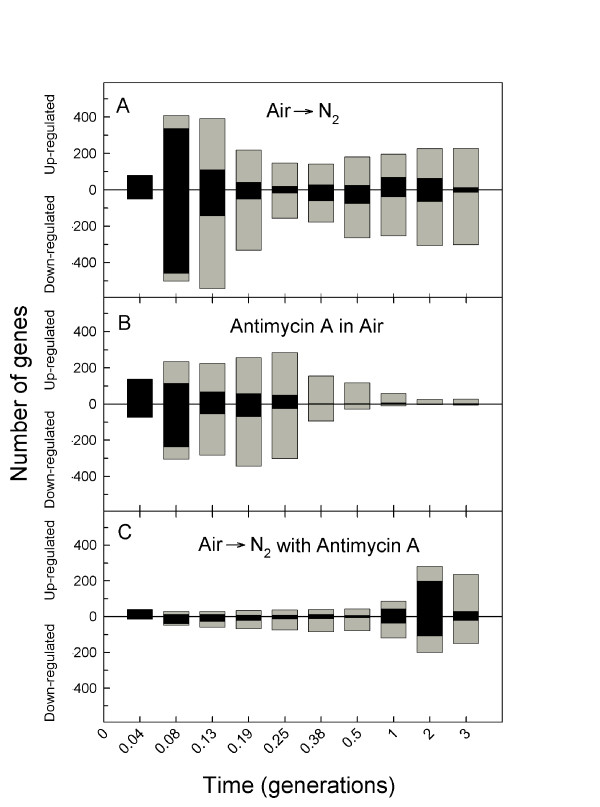
**Dynamics of gene induction and repression in response to anaerobiosis (A), antimycin A treatment in air (B) and subsequent anaerobiosis in the presence of antimycin A (C)**. The number of genes that responded significantly (*P *≤ 0.01) at each time point is shown. Genes are divided into those that were up-regulated from those that were down-regulated. Black bars indicate genes that significantly responded for the first time at the indicated time point whereas gray bars indicate genes that had already been identified at an earlier time point yet continued to be differentially expressed as compared to the control. The combined height of the black and gray bars is the total number of genes at each time point that were differentially expressed as compared to the aerobic control.

Figure 1 compares the overall dynamics of each response expressed as the number of genes that were differentially expressed in each sample. Note that black bars indicate genes that responded significantly for the first time and gray those that had already responded at a previous time point yet continued to be differentially expressed with respect to the control. From this figure it is clear that the transcriptomic response to anaerobiosis in galactose medium is biphasic (Figure 1A) as was previously observed [6]. The first phase is comprised of a large set of genes (> 900) that exhibit an acute (0.04 – 0.08 generations) yet transient (< 0.25 generations) response. After a considerable delay (≥ 0.25 generations), a second, smaller set of genes (> 400) is then differentially expressed, most for the duration of anaerobiosis. In comparison, the transcriptomic response to antimycin A treatment in air (Figure 1B) is monophasic and, as anticipated, similar to the dynamics of the acute phase of the anaerobic response. Peak numbers of newly responding genes (black bars) appear at 0.08 generations, the same as for the acute anaerobic response (Figure 1A).

Comparison of Figure 1C with 1A shows the presence of antimycin A drastically alters the anaerobic response; the large numbers of transiently responding genes observed in Figure 1A are conspicuously absent, leaving only those that respond after a substantial delay (2 generations). Interestingly, the dynamics of this response is remarkably similar to that elicited by anoxia in catabolite-repressed cells [5,6]. Taken together these results suggest that the acute, transient transcriptomic response observed in Figures 1A and 1B is evoked by the inhibition of respiration, whether by chemical means (antimycin A) under normoxic conditions (Figure 1B) or the rapid removal of oxygen from respiratory competent cells (Figure 1A), whereas the delayed, chronic phase (Figures 1A and 1C) is invoked by oxygen deprivation.

**Figure 2:**
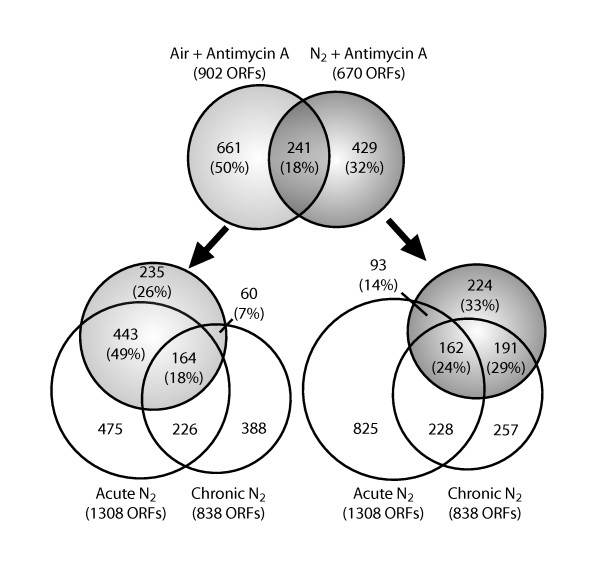
**Comparison of genes that comprise the acute, transient phase of the anaerobic response with those that respond to antimycin A treatment in air and subsequent anaerobiosis**. Genes that responded within the first 0.25 generations of anaerobiosis were classified as acutely responding (acute N_2_) whereas those that responded after this period were classified as chronically responding (chronic N_2_) (see Figure 1A for phase dynamics). Genes that comprise each of these classes are compared to those that responded to the antimycin A treatments (light gray for air and darker gray for N_2_). The area of the circles is scaled to the number of genes (ORFs), and the percentage of genes found in each phase is indicated in the parentheses.

To further explore the composition of these networks, we divided the anaerobic gene set into those that responded acutely (≤ 0.25 generations) and those that responded more chronically (> 0.25 generations) and examined their overlap with those that responded to the antimycin A treatments. Figure 2 shows 67% of those that responded to the antimycin A treatment in air (light gray circle, left) exhibited an acute anaerobic response (bottom left) whereas 53% of those that responded to the subsequent shift to anaerobiosis (dark gray circle on right) exhibited a delayed anaerobic response (bottom right). Although the distinction between the acute and chronic phases is not absolute (Figure 2 open circles, lower portion), what is clear is that many of the same genes respond acutely to either anaerobiosis or antimycin A treatment in air. Moreover, the acute response is abolished by adding antimycin A well before (3 generations) shifting the cells to anaerobiosis. To next determine if the same transcriptional networks respond to these treatments, we separately clustered the temporal responses.

### Gene network discovery

**Figure 3:**
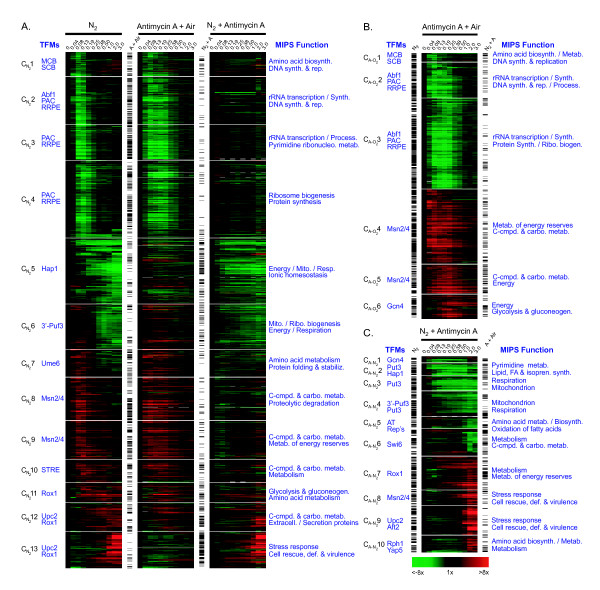
**Transcript heat maps and statistical comparisons of genes that significantly responded to anaerobiosis (A), antimycin A treatment in air (B), and subsequent anaerobiosis in the presence of antimycin A (C)**. The temporal profiles of genes whose transcript levels responded significantly (*P *≤ 0.01) to each treatment were clustered separately using Kohonen's SOM algorithm with 1D string topology and standard correlation as the distance metric. Appropriate K values for gene network discovery were determined by evaluating CS and FCS using a range of K values (2 – 30) (see "Materials and Methods"). Figure 3A compares the response of all genes that significantly responded to anaerobiosis (N_2_, far left) to their response to antimycin A treatment in air (middle panel) and to subsequent anaerobiosis in the presence of antimycin A (right panel). Note that only those genes denoted by a black bar responded significantly to the indicated treatment (A + Air = antimycin A under aerobiosis, N_2 _+ A = antimycin A under anaerobiosis, and N_2 _= anaerobiosis). The blue text is an abridged list of transcription factor motifs (TFMs, left) and MIPS functional categories (MIPS Function, right) that were significantly (*P *≤ 0.01) overrepresented in each gene cluster. Panel B shows the clustering results for all genes that significantly responded to antimycin A treatment in air while panel C shows that for those significantly responded to anaerobiosis in the presence of antimycin A. The time scale is the number of generations after the indicated treatment.

Gene networks were recovered using a novel bioinformatic approach [5,6] that uses SOM to cluster the temporal profiles and then determines an appropriate level of granularity (number of clusters) by choosing that which results in the *least likely *configuration of transcription factor binding sites (TFMs) among gene clusters as compared to random chance alone [see Additional files 1 and 2 and reference [6] for further details]. For the anaerobic data set, the clustered expression profiles are shown in Figure 3A (left heat map; anaerobic clusters ${\text{C}}_{{\text{N}}_{\text{2}}}$1 – 13). The figure also includes an abbreviated list of significantly (*P *≤ 0.01) enriched TFMs (blue text to the left of each heat map) and overrepresented (*P *≤ 0.01) MIPS functional categories (right). Complete results, including statistics and gene-to-cluster membership, are provided in Additional files 3, 4, 5, 6. To facilitate direct comparisons of the two antimycin A treatments to anaerobiosis, Figure 3A not only shows the clustered expression profiles of genes that responded to anaerobiosis (N_2_, left heatmap) but the response of these same genes to antimycin A treatment in air (Antimycin A + air, middle) and to a shift to anaerobiosis after pre-treatment with antimycin A (N_2 _+ antimycin A, right). Note that only those genes that are indicated by the black bars to the left of each heat map responded significantly (*P *≤ 0.01) in the presence of inhibitor. Finally, Figure 3B shows the clustered expression profiles and recovered gene networks for the 901 genes that responded significantly to antimycin A treatment in air (${\text{C}}_{{\text{A-O}}_{\text{2}}}$1 – 6) and Figure 3C the same for the 669 genes that responded to the subsequent shift to anaerobiosis (${\text{C}}_{{\text{A-N}}_{\text{2}}}$1 – 10).

Comparison of the left (anaerobic) and middle (antimycin A + air) heat maps in Figure 3A shows that most of the genes that transiently responded to anaerobiosis (${\text{C}}_{{\text{N}}_{\text{2}}}$1 – 4 and ${\text{C}}_{{\text{N}}_{\text{2}}}$7 – 9) responded in a similar manner to antimycin A treatment in air. In contrast, few of those that exhibited a delayed, chronic response (${\text{C}}_{{\text{N}}_{\text{2}}}$5 – 6 and ${\text{C}}_{{\text{N}}_{\text{2}}}$10 – 13) were differentially expressed in response to inhibitor treatment in air. These trends are almost totally reversed for the N_2 _+ antimycin A treatment (right heatmap in Figure 3A) as can be easily seen by comparing the distribution of the significance indicators (black bars) among gene clusters for each treatment.

In regard to the recovered gene networks, comparison of Figure 3A with 3B indicates the same or similar TFMs and MIPS functional categories were enriched in clusters of genes that transiently responded. From this and previous studies of the anaerobic response [5,6], a common picture of gene network activity emerges. For example, both anaerobiosis and antimycin A treatment in air result in the transient down-regulation of MCB- and SCB-regulated networks (${\text{C}}_{{\text{N}}_{\text{2}}}$1 and ${\text{C}}_{{\text{A-O}}_{\text{2}}}$1) involved in processes associated with the G1/S transition of the cell cycle (e.g., DNA synthesis & replication). In addition, Abf1 and PAC/RRPE-associated networks (${\text{C}}_{{\text{N}}_{\text{2}}}$2 – 4 and ${\text{C}}_{{\text{A-O}}_{\text{2}}}$2 & 3) involved in energetically costly programs of rRNA transcription/processing, translation, and ribosomal biogenesis are apparently also down-regulated. Finally, both treatments apparently result in the transient induction of Msn2/4-regulated networks (${\text{C}}_{{\text{N}}_{\text{2}}}$8 – 10 and ${\text{C}}_{{\text{A-O}}_{\text{2}}}$4 & 5) involved in carbohydrate and reserve energy (trehalose and glycogen) metabolism/transport and autophagy.

In contrast, networks that more chronically responded to anaerobiosis, in particular those with identifiable heme-responsive transcription factor binding sites (eg., Hap1 for ${\text{C}}_{{\text{N}}_{\text{2}}}$5, and Rox1 and Upc2 for ${\text{C}}_{{\text{N}}_{\text{2}}}$10 – 13), failed to respond to the antimycin A treatment in air (Figure 3B) but were recovered from clustering the response to antimycin A treatment in N_2 _(Hap1 in ${\text{C}}_{{\text{A-N}}_{\text{2}}}$2, Rox1 in ${\text{C}}_{{\text{A-N}}_{\text{2}}}$7, and Upc2 in ${\text{C}}_{{\text{A-N}}_{\text{2}}}$9 in Figure 3C). A comparison of these heatmaps shows the response of these networks is kinetically very similar in the two anaerobic datasets. Similarities included a slightly delayed (ca. 0.08 generations) yet chronic down-regulation of Hap1-regulated networks involved in respiration and mitochondrial energy production (${\text{C}}_{{\text{N}}_{\text{2}}}$5 and ${\text{C}}_{{\text{A-N}}_{\text{2}}}$2) and delayed yet chronic down-regulation of networks involved in mitochondrial ribosomal biogenesis (${\text{C}}_{{\text{N}}_{\text{2}}}$6 and ${\text{C}}_{{\text{A-N}}_{\text{2}}}$4) that were significantly enriched for 3'-Puf3 sites. Both treatments also resulted in the delayed yet chronic de-repression of Rox1-regulated networks (${\text{C}}_{{\text{N}}_{\text{2}}}$11 – 13 and ${\text{C}}_{{\text{A-N}}_{\text{2}}}$7) involved in carbohydrate import/utilization and redox balance and delayed activation of Upc2-regulated ones (${\text{C}}_{{\text{N}}_{\text{2}}}$11 – 13 and ${\text{C}}_{{\text{A-N}}_{\text{2}}}$9) involved in sterol and cell wall homeostasis. Although there are differences in terms of specific transcription factor binding sites and functional categories enriched from clustering the two anaerobic datasets, it is clear that the networks that more chronically respond to anaerobiosis are not affected by the presence of the respiratory inhibitor.

Overall, it is apparent from these comparisons that the normoxic addition of antimycin A to respiratory competent cells and oxygen deprivation elicits a kinetically similar response in the same gene networks (MCB/SCB, Abf1/PAC/RRPE and Msn2/4). Moreover, pretreatment of aerobic cells with antimycin A abrogates the transient response of a large number of networks that acutely respond to anoxia yet has not affect heme-regulated ones (e.g., Hap1, Rox1 and Upc2) that more chronically respond. These results show that the acute and chronic phases of the anaerobic response in catabolite non-repressed (galactose) cells are separable and, thus, are likely triggered by different sensing and signaling pathways: the acute transient phase from the respiratory inhibition and, we hypothesize, associated changes in cellular energetics and/or growth rate, and the chronic phase from oxygen deprivation. This interpretation is further corroborated by previous studies that show this transient "stress response" to anaerobiosis is absent in catabolite-repressed (glucose) cells [5]. Under both conditions, respiration is inhibited or repressed during the transition to anaerobiosis: here by chemical means and in previous studies by the presence of glucose. Thus, in both cases, the aerobic to anaerobic transition proceeds rather smoothly, that is without a major disruption in catabolism or measured growth rate as the cells ferment the available carbon source in both the aerobic and anaerobic phases. In contrast, cells grown on galactose in the absence of respiratory inhibitor are forced to switch from respiro-fermentative to strictly fermentative metabolism and, in so doing, undergo a substantial decrease in growth rate (here from a 2.4 h mass doubling time to 4 h). Although several recent studies have suggested the changes in the activity of these general stress-responsive gene networks result simply from changes in growth rate [7-9], given their function and the fact they only transiently respond and are not differentially expressed among treatments where the grow rate differs nearly 2-fold (aerobiosis vs. anaerobiosis) suggests they may be part of a systems level response for balancing energetic supply and demand and coordinating entry into the cell cycle in response to the abrupt cessation of respiration.

## Discussion

This study has revealed novel insight into the transcriptomic response referred to as the "common environmental response" (CER) [4] or "environmental stress response" (ESR) [2] and differentiates between networks that respond to respiratory inhibition and those that respond to oxygen deprivation. First, we show that the acute inhibition of respiration by either oxygen deprivation (anoxia) or chemical means (antimycin A) in catabolite non-repressed conditions evokes a transient ESR/CER-like response. Gene network analyses suggest that changes in the activity of Fhl1 and PAC/RRPE-associated factors result in the transient down-regulation of genes involved in energetically costly programs of ribosomal biogenesis, protein synthesis, and rRNA transcription/processing. Simultaneously, transient changes in the activity of the SBF (Swi4-Swi6) and MBF complexes (Mbp1-Swi6) result in the down-regulation of genes involved in late G1 and the G1/S transition of the cell cycle and a predicted delay in its progression as mass and energy are assessed before committing to another round. At the same time Msn2/4-regulated networks involved in carbohydrate import/utilization and reserve energy metabolism are transiently activated.

When viewed together it seems clear that the transient changes in the activity of these gene networks may be required for balancing energy supply and demand and regulating entrance into the cell cycle. Functional gene network analyses suggest this program includes simultaneously bolstering catabolic potential, regulating reserve energy supplies (trehalose and glycogen), and sparing energetic demand by down-regulating early steps in the biogenesis of the cytoplasmic ribosomes. Given that changes in the activity of these gene networks are not observed when respiration is inhibited well before (3 generations) the aerobic-to-anaerobic transition, we hypothesize these networks may respond to the acute decrease in energy status that accompanies respiratory inhibition as it is clear they do not respond directly to the change in oxygen availability. This conclusion is further supported by previous genomic comparisons of the anaerobic shift in catabolite-repressed and non-repressed cells [5,6], which showed that these networks also fail to respond during the aerobic-to-anaerobic transition when respiration is inhibited by the presence of glucose and, thus, when the transition precedes with no change in growth rate or energetic status. During the review of this manuscript, an additional genomic study examining the switch to fermentation also concluded that the mere depletion of oxygen does not evoke a stress response [11].

After a substantive delay (≥ 2 generations), more chronic changes in gene network activity were observed under anaerobiosis that were not observed under aerobiosis in the presence of antimycin A. Network analyses show these included the chronic down-regulation of Hap1- and Hap2/3/4/5-regulated ones involved in mitochondrial functions as well as Rox1-regulated ones involved in redox regulation and carbohydrate usage. The last networks to respond (≥ 1 generation) were Upc2-regulated ones involved in cell wall functions and sterol homeostasis. These same networks responded with similar kinetics to anoxia in the presence of antimycin A. An extensive body of previous research (reviewed in [12-14]) has shown that changes in the activity of these *trans*-acting factors result from decreased heme levels as a result of the loss of oxygen for its synthesis and, thus, it is not surprising that these networks fail to respond to antimycin A treatment under normoxic conditions. Others include those involved in mitochondrial ribosomal biogenesis that have apparently lost their 5' ancestral *cis*-regulatory sites and appear to be regulated post-transcriptionally by Puf3 [15]. Although some genes were found to respond in a unique manner to each treatment, clustering analyses reveal the same or similar gene networks respond to oxygen deprivation both the presence and absence of inhibitor.

Given that both oxygen deprivation and antimycin A poisoning require metabolic retooling of mitochondrial functions, it is reasonable to postulate that many of the observed changes in gene expression result from retrograde signaling [16]. The retrograde response signals mitochondrial dysfunction to the nucleus and causes changes in the expression of genes associated with peroxisomal activities and anaplerotic pathways that mitigate the loss of the tricarboxylic acid cycle activity [17]. This response also correlates metabolism with stress responses, chromatin-dependent gene activation, and genome stability in yeast aging [18]. Not surprisingly, many of the known RTG-dependent genes responded to antimycin A treatment in air [19]. However, there was surprisingly little overlap (21%) between the genes identified here to respond to antimycin A treatment and those from Epstein et. al's study [17], differences that most likely can be attributed to the time points that were sampled in each study, array platforms and differences in experimental conditions. Rather, it is clear that the gene networks that respond to anoxia as well as antimycin A treatment are those involved in the general cellular response to stress.

Is the acute response to respiratory inhibition an "environmental stress response"? While it is common practice to look for congruence in lists of differentially expressed genes among various treatments to answer this question, such comparisons are more meaningful and robust when comparing functional groups and transcriptional units defined by common *cis*-regulatory sequences statistically enriched in the clustered genes. Even when identical experimental treatments are compared, differences in strain backgrounds, media, time courses, expression platforms, statistical criteria, and experimental variation can result in frighteningly little congruence among gene sets. For example, when we compare our transiently responding genes to those that comprise the ESR [2], there is only modest (40%) overlap. However, after performing SOM clustering of the ESR data and calculating FCS and CS as we did for our data sets, we recovered eight clusters of transiently up-regulated genes and four clusters of transiently down-regulated genes in which the predominant *cis*-regulatory sequences and functional categories were nearly the same as in our study. These include rapidly repressed networks involved in aminoacyl-tRNA synthesis and protein synthesis that are enriched for SCB, Swi6 and Abf1 binding sites, and several clusters of transiently repressed genes involved in ribosomal biogenesis, rRNA transcription/processing and/or protein synthesis that are significantly enriched for Fhl1, Rap1, PAC, RRPE, and/or Abf1 sites. Those that were transiently induced are predominated by Msn2/4 and/or Nrg1 sites and enriched for categories of rescue and defense, the stress response, metabolism of energy reserves and peroxisome function. Thus, from a network comparison it is clear that the same or similar gene networks respond to respiratory inhibition as they do to a large number of other environmental challenges.

### Functional Attributes of the General Stress Response

The general stress response was first postulated to explain the phenomenon of cross-protection, wherein exposure to a non-lethal dose of one stress protects against a potentially lethal dose of another, often seemingly unrelated, stress [20-25]. It is now clear that this cross protection is afforded by the transcriptional up-regulation of a common set of general stress-responsive genes involved in diverse cellular functions, including reserve energy regulation, carbohydrate metabolism, protein folding and degradation, oxidative stress defense, autophagy, cytoskeletal reorganization, DNA-damage repair, and other processes [2,4]. However, the degree of cross-protection varies depending on the stresses and it is not always reciprocal, indicating that stress-specific responses are required for full protection from a specific stressor. Stress resistance has also been shown to occur following nutrient deprivation and stationary phase [2,26-28]. A number of different signaling pathways acting in response to specific stressors have been shown to control these stress-responsive genes. Signaling pathways implicated in coordinating the response include the protein kinase C (PKC)-mitogen-activated protein (MAP) kinase pathway following secretion defects and cell wall damage [29-32], the MEC1 pathway following DNA damage [33], and the Ssk1/Ste11-dependent pathways and high osmolarity glycerol (HOG)-MAP kinase cascade following osmotic stress [1,34]. Pathways involved in suppressing the response include the target of rapamycin (TOR) pathway [35], the Snf1 protein kinase pathway [36], and the PKA-MAP kinase pathway [37-39]. Although seemingly complex, such a multiplex of signaling cascades is likely required for dictating specificity in the cellular response to an environment in which a multitude of different parameters (e.g., temperature, osmolarity, pH, O_2 _and other nutrients) can change simultaneously.

From functional analyses of the response, it is clear that a rapid change in steady-state conditions (environmental or physiological) prepares the cell to meet a myriad of unforeseen challenges. From a systems level viewpoint, the cell must coordinate energy metabolism with growth rate, the cell cycle, and any specific requirements dictated by the environmental conditions. Given the gene networks that respond, it would appear that at its core is the sparing of energetic demand by rapidly down-regulating the expression of a large set of genes (~600) involved in energy consuming pathways. Simultaneously, supplies may be bolstered by up-regulating key genes involved in import/utilization of primary and secondary carbon substrates and presumably recycling cellular components through autophagy. More complex changes are observed for genes involved in reserve energy stores (trehalose and glycogen), which presumably reflect appropriate parsing of supplies for cell-cycle function and stress mitigation while preserving a modicum of reserve capacity. In addition to changes in gene expression, many of these latter genes are regulated post-transcriptionally.

In terms of potential energetic sparing measures, more than 70% of the characterized genes [40,41] whose expression is down-regulated in the ESR are involved in protein synthesis, including genes required for ribosome synthesis and processing, RNA polymerase I- and III-dependent transcription, and protein translation. Thus, it should be of no surprise that these include some of the most abundant and shortest-lived mRNAs, namely genes for ribosomal proteins (RP genes) and rRNA transcription. RP genes alone account for about 60% of all transcription-initiation events in rapidly growing yeast cells [42] and a remarkable 90% of all mRNA splicing events. Temporal analyses suggest rapid transcriptional deactivation or silencing of these genes followed by mRNA decay given that the decline in their transcript levels tracks well with their estimated half-lives. Predictable yet less dramatic differences in the expression of these, as well as most other genes that comprise the common stress response, have been shown to result from simply varying the growth rate [7], providing further evidence that their expression is in step with the physiological status of the cell.

In nearly all conditions examined except nutrient starvation [2], the stress response has shown to be transitory, with maximal transcriptional changes frequently observed between 15 and 30 min and a diminishing response after 60 min. Although, multiple interpretations have been provided (e.g., see discussions in [2,4,43]), a simple hypothesis that fits with the transcriptomic data is that all of these environmental challenges, whether perturbations that temporally disrupt catabolic pathways or ones that require rapidly up-regulating costly programs for combating a specific stressor, require the immediate expenditure of sufficient energy as to require the suspension of other cellular operations, such as general protein synthesis. Obviously, such a hypothesis needs to be corroborated with careful measurements of cellular energetics, measurements that are the focus of ongoing studies in the laboratory. After the initial energetic drain, it would appear that a new balance is quickly achieved and cells resume their normal activities given these genes do not continue to be differentially expressed except under nutrient starvation conditions. Moreover, such an interpretation suggests the response is graded according to the degree to which the energetic status of the cell is perturbed, a hypothesis that is directly testable and the subject of additional ongoing experiments in our laboratory.

Finally, several recent studies have suggested that many of the genes that have been previously characterized as "stress responsive" are perhaps more appropriately labeled "growth-rate responsive" [7-9]. In a serious of elegant, nutrient-limited chemostat studies, these authors have shown the expression of most of the genes comprising the ESR is in-step with steady-state growth rate. Thus, rather than responding to the stress directly, perhaps they are responding to a change in growth rate secondary to the stress [8,9]. To determine if the genes we identified are correlated with growth rate, we divide the clustered expression profiles of the anaerobic dataset into groups of transiently and chronically responding ones and then further into repressed and induced sets. Using the online utility  developed by Brauer *et al. *2008 [9] we plotted the distribution of growth-rate slopes for these sets. As shown in Additional file 7, those that are transiently expressed are highly correlated with growth rate whereas those that were more chronically expressed are not. Given this, it is also possible to infer the instantaneous growth rate using these transiently expressed genes [9], which in our datasets suggests a maximal slowing of growth between 0.13 and 0.19 generations (ca. 30 min) in response to N_2 _and antimycin A treatment in air and complete recovery by about 0.5 generations (ca. 2 h). If changes in the expression of these genes are a direct result of differences in growth rate, what is puzzling here is the fact they are not differentially expressed between the strictly fermentative phase, in which the mass doubling time is 4 h, and the respiro-fermentative phase, in which the mass doubling time is 2.4 h. Rather the response is strictly transitory. What's more is that inhibiting galactose-dependent respiration limits the rate at which ATP can be generated (Q_ATP_) and, thus, it is hard to see how acute changes in cellular energetics cannot be at the core of this response. In other words, that it is the acute change in cellular energetics that initiates the response resulting in the change in growth rate, a hypothesis that is being tested with on-going experiments in our laboratory.

In summary, all eukaryotic cells have necessarily evolved mechanisms for effectively dealing with fluctuations in physical, chemical, and physiological parameters. Even within the relatively protected confines of multicellular organisms, cells experience fluctuations in osmolarity, oxygen and reactive oxygen species as well as various nutrients and noxious substances. Comparative studies have shown that many of the factors important for regulating the stress response, including both signaling pathways (e.g., MAPK) and transcription factors (e.g., AP-1), are conserved from yeast to humans. Understanding the factors that influence this response has important implications for pathophysiological conditions, such as heart disease, tumorigenesis, various metabolic syndromes, and aging, all of which contribute to stress at the cellular level. Here we set forth a hypothesis that at its core is the regulation of cellular energetics via an as yet unidentified metabolic signal. Such internal signals are being actively studied in bacteria [44] but comparatively little work has been done in eukaryotes. Two, partially redundant, serine/threonine kinases containing PAS domains, Psk1 andPsk2, which coordinately regulate protein synthesis and carbohydrate metabolism in response to nutritional status of the cell, have been implicated in signaling in yeast. Although a small molecule/metabolite is presumed to trigger this response by interacting with the PAS domain, it has not been identified [45,46]. Variants of this system are found from yeast to mammals [47-49]. Thus, future studies with such factors will be of great interest to unravel both the sensing and signaling aspects of this conserved stress response program.

## Methods

### Media and growth conditions

The *S. cerevisiae *strain JM43 (*MATα leu2-3,112 his4-580 trp1-289 ura3-52 [ρ +]*)[50] was used. Liquid pre-cultures were grown in a semi-synthetic galactose medium containing Tween 80, ergosterol, and silicon antifoam (SSG-TEA) [51] with shaking (300 rpm) for 3 – 4 days prior to inoculating a NewBrunswick BioFlo III fermentor (3.5 l working volume) [52]. Batch fermentor cultures were inoculated with a volume of pre-culture to allow for at least six generations of aerobic growth before reaching a cell density of ≈ 1 Klett unit. A control sample was then harvested and 1 μM antimycin A (final concentration; Sigma, St. Louis, MO) was added to the medium. Ten samples were collected (0.04, 0.08, 0.13, 0.19, 0.25, 0.38, 0.5, 1, 2, and 3 generations) over the next three generations of aerobic growth. The sparge gas (1.2 vol. of gas/vol. of medium per min) was then switched from air to 2.5% CO_2 _in O_2_-free N_2 _and the same generation-specific samples were taken over three generations of anaerobic growth. Dissolved O_2 _was controlled and monitored as described previously [52]. Two batch fermentor experiments, in which every other time point was sampled, were conducted to complete the entire time series, which was repeated in triplicate. Cells were harvested using a vacuum filtration apparatus onto filters as described previously [53]. The filtered cells were washed with either sterile de-oxygenated or oxygenated water (as appropriate), flash-frozen in liquid N_2 _within one minute of initiating the sampling, and stored at -80°C for later RNA isolation.

### RNA extraction, cDNA synthesis, and microarray hybridization

Total RNA was extracted from the filtered cells using hot phenol as described previously [53]. Thirty μg of total RNA was used for first-strand cDNA synthesis, and microarray target preparation was performed as described elsewhere [53]. A reference design was used for hybridizing the custom 70-mer oligonucleotide microarrays [5]. The reference consisted of a pool of equal masses of RNA from a 24-h anaerobic culture and a 4-h aerobic culture harvested after prior exposure to 24 h of anaerobiosis. Microarray hybridizations, washing, and scanning were conducted as described previously [5].

### Statistical analyses and data clustering

GenePix Pro software (v4.1, Axon, Union City, CA) was used for spot identification and fluorescence intensity quantification. Data normalization and statistical analyses were performed as described previously [5] using SAS (v9.1, SAS Institute, Cary, NC). After normalization, separate ANOVAs (SAS MIXED procedure with repeated measures) were used to identify genes whose transcript levels responded significantly to three different treatments: (A) anaerobiosis, using data published previously from our laboratory [6]; (B) antimycin A treatment during aerobiosis; and (C) anaerobiosis in the presence of antimycin A. The same generation-specific sampling regime was used for each treatment (0 [control], 0.04, 0.08, 0.13, 0.19, 0.25, 0.38, 0.5, 1, 2, and 3 generations). A step-down Bonferroni post hoc *P*-value adjustment was used to minimize the false discovery rate.

After identifying genes that responded significantly to each treatment, the temporal profiles were separately clustered using Kohonen's self-organizing map (SOM) algorithm [54]) with 1D-string topology and standard correlation as the distance metric. To determine an appropriate K value (cluster number) for gene network recovery, two quality assessment metrics were calculated from the results obtained using a range of K values (from 2 to 30). Consensus Share (CS) is the percentage of genes that were clustered exactly the same over 10 replicate runs of the SOM algorithm (an indicator of clustering robustness), and the Feature Configuration Statistic (FCS) is the probability that the observed configuration of a transcription-factor motif (TFM) among gene clusters arose by chance alone from its multinomial distribution dictated by cluster sizes [5]. For FCS, we assessed the configuration of 2,892 transcription-factor consensus binding sequences or motifs (TFMs) compiled from both experimental and comparative phylogenomic analyses [see Additional file 1]. To narrow the range of potentially interesting K values, we first eliminated those with a CS value < 0.9. We then calculated FCS q values [55] on a K-by-K basis and eliminated any TFMs whose value was > 0.01 for all remaining K values. We then calculated an average FCS P value for all remaining TFMs as a function of K. The clustering result (K value) for each treatment that resulted in the lowest average FCS P value was chosen for gene network analysis. In addition to examining the configuration of consensus binding sequences among gene clusters (FCS *P *value), and calculating their hypergeometric enrichment *P *values within each cluster, we also used MDscan [56] to identify overrepresented sequences in each of the gene clusters that may not be included in our TFM list. For training, we used a set of ≈ 30 expression profiles, but no more than 50% of any cluster, that were closest to the mean expression profile in each cluster. FunSpec [57] was used to calculate hypergeometric *P *values for enriched MIPS functional categories in each gene cluster or for subgroups of genes identified with MDscan.

All microarray data from this study have been deposited in NCBI Gene Expression Omnibus (accession numbers GSE3705 and GSE3706). The anaerobic dataset to which the antimycin A treatments are compared is a subset of accession number GSE2246.

## Authors' contributions

L-CL helped grow the strains, carried out the microarray studies, conducted the statistical and clustering analyses, and helped draft the manuscript. MTK assisted with the microarrays and the statistical and clustering analyses. PVB participated in the design of the study, the growth of strains, collection of RNA, interpretation of the clustering analyses, and drafting the manuscript. KEK conceived the study, participated in its design and coordination, and helped to draft the manuscript. All authors read and approved the final manuscript.

## Supplementary Material

Additional file 1 **Transcription-factor consensus binding sequences (TFMs).** The table lists the 2,892 transcription-factor consensus binding sequences (TFMs) compiled from both experimental and comparative phylogenic studies that were used for calculating the feature configuration statistic (FCS). Click here for file

Additional file 2 **Assessment of clustering quality using the feature configuration statistic (FCS) and consensus share (CS) for genes that significantly responded to anaerobiosis (N_2_), antimycin A treatment in air (antimycin A + air), and anaerobiosis in the presence of antimycin A (N_2 _+ antimycin A).** The temporal profiles of genes that responded significantly (*P *< 0.01) to each treatment were separately clustered 10 times using Kohonen's SOM algorithm with 1-D string topology and Pearson correlation as the distance metric. The average FCS *P *value (solid line, left ordinate) for 2,892 transcription-factor consensus binding sequences (TFMs) and CS (dotted line, right ordinate) are plotted as a function of cluster number (K). Click here for file

Additional file 3 **Abridged list of TFMs, MDscan sequence logos, and MIPS functional categories significantly enriched from clustering the genomic responses to each treatment.** The tables provide an overview of consensus sequence motifs (TFMs), MDscan sequence logos, and MIPS functional categories that were significantly (p < 0.01) enriched from clustering the genomic responses to anaerobiosis (A), antimycin A treatment under normoxia (B), and antimycin A treatment under normoxia (C). See additional files 4, 5, 6 for unabridged listings. Click here for file

Additional file 4 **Unabridged list of TFMs and MIPS functional categories that were significantly (*P *< 0.01) enriched from clustering the 1754 genes that were differentially (*P *< 0.01) expressed in response to anaerobiosis in galactose medium.** The data are for the wild-type strain over 3 generations of anoxia. SOM clustering was used with K = 13. Genes that are contained in each of the clusters presented in Figure 3A in the manuscript are provided in the "Gene-Cluster Membership" worksheet. Click here for file

Additional file 5 **Unabridged list of TFMs and MIPS functional categories that were significantly (p < 0.01) enriched from clustering the 901 genes that were differentially (p < 0.01) expressed in response antimycin A treatment under normoxia in galactose medium.** The data are for the wild-type strain over 3 generations following antimycin A treatment in air. SOM clustering was used with K = 6. Genes that are contained in each of the clusters presented in Figure 3B in the manuscript are provided in the "Gene-Cluster Membership" worksheet. Click here for file

Additional file 6 **Unabridged list of TFMs and MIPS functional categories that were significantly (p < 0.01) enriched from clustering the 669 genes that were differentially (p < 0.01) expressed in response antimycin A treatment under anoxia in galactose medium.** The data are for the wild-type strain over 3 generations of anoxia following a 3-generation treatment with antimycin A in air. SOM clustering was used with K = 10. Genes that are contained in each of the clusters presented in Figure 3C in the manuscript are provided in the "Gene-Cluster Membership" worksheet. Click here for file

Additional file 7 **Distribution of regression slopes for genes that were acutely repressed (Figure 1), chronically repressed (Figure 2), acutely induced (Figure 3), and chronically induced (Figure 4) in response to anaerobiosis.** The graphs show the distribution of growth-rate slopes for specific gene clusters from the anaerobic dataset (black) in comparison to ESR-induced (red) and ESR-repressed genes (green). The graphs were generated using the online utility  developed by Brauer *et al. *2008 [9]. Click here for file
