# Supplementary figures and images for: Comparison of the transcriptomic "stress response" evoked by antimycin A and oxygen deprivation in saccharomyces cerevisiae
Source: BMC Genomics. 2008 Dec 23;9:627. doi: 10.1186/1471-2164-9-627 (PMC2637875; doi:10.1186/1471-2164-9-627)

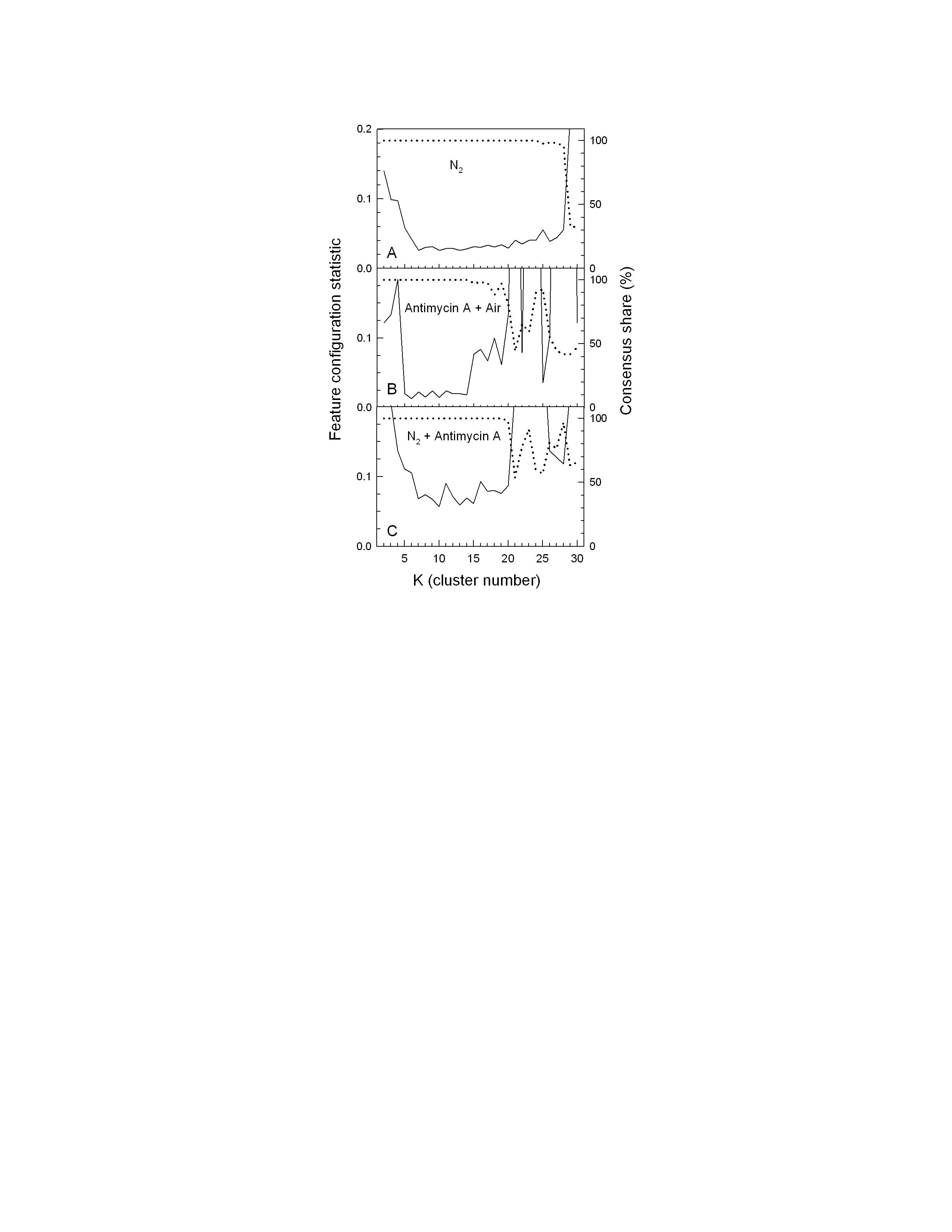

Supplement: Additional file 2 — Assessment of clustering quality using the feature configuration statistic (FCS) and consensus share (CS) for genes that significantly responded to anaerobiosis (N2), antimycin A treatment in air (antimycin A + air), and anaerobiosis in the presence of antimycin A (N2 + antimycin A). The temporal profiles of genes that responded significantly (P < 0.01) to each treatment were separately clustered 10 times using Kohonen's SOM algorithm with 1-D string topology and Pearson correlation as the distance metric. The average FCS P value (solid line, left ordinate) for 2,892 transcription-factor consensus binding sequences (TFMs) and CS (dotted line, right ordinate) are plotted as a function of cluster number (K). [file 1471-2164-9-627-S2.jpeg]
