# Supplementary material for: Comparison of the transcriptomic "stress response" evoked by antimycin A and oxygen deprivation in saccharomyces cerevisiae
Source: BMC Genomics. 2008 Dec 23;9:627. doi: 10.1186/1471-2164-9-627 (PMC2637875; doi:10.1186/1471-2164-9-627)
Supplement: Additional file 3 — Abridged list of TFMs, MDscan sequence logos, and MIPS functional categories significantly enriched from clustering the genomic responses to each treatment. The tables provide an overview of consensus sequence motifs (TFMs), MDscan sequence logos, and MIPS functional categories that were significantly (p < 0.01) enriched from clustering the genomic responses to anaerobiosis (A), antimycin A treatment under normoxia (B), and antimycin A treatment under normoxia (C). See additional files 4, 5, 6 for unabridged listings. [file 1471-2164-9-627-S3.doc]

**TABLE 1**. Selected list of enriched consensus sequence motifs (TFMs), MDscan sequence logos, and MIPS functional categories in clusters*^a^* of genes differentially expressed in response to anoxia in galactose medium.

| **Cluster** | **Gene**  **no*.*** | **TFM** | **Reference(s)** | **Share (%)** | ***P*-value*^b^***  **(set/genome)** | **MDscan motif logo (corr.)**  **(freq. & score)** | **MIPS functional category enrichment** | ***P*-value*^c^***  **(genomic)** |
| --- | --- | --- | --- | --- | --- | --- | --- | --- |
| C_N2_ 1 | 83 | SWI4 | [1-6] | 70 | 5.2 / 3.5 | 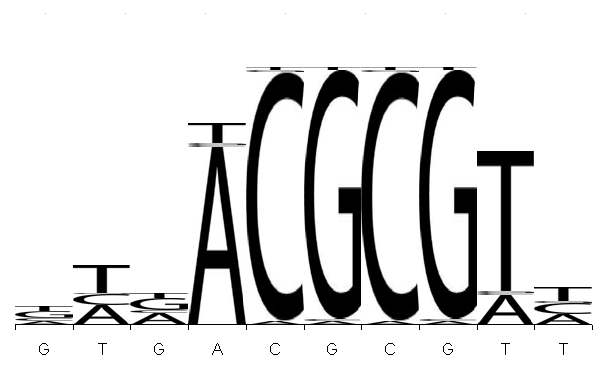  MBP1^[2]^ (0.99), MCB ^[7]^ (0.99)  (41/83 genes, 3.9) | Amino acid biosynthesis / Metabolism | ≥9.1 |
|  |  | MCB | [8, 9] | 50 | 5.5 / 5.4 |  | Metabolism | 5.7 |
|  |  | SCB | [2, 8, 9] | 40 | 2.2 / 2.2 |  | DNA synthesis & replication / Process.  (*for MDscan Motif Genes, P ≥ 7.1*) | ≥ 4.5 |
|  |  | SWI6 | [1, 2, 4] | 40 | 5.1 / 4.0 |  | Cell cycle and DNA processing  *(for MDscan Motif Genes, P = 6.6* ) | 3.4 |
| C_N2_ 2 | 164 | ABF1 | [1-5, 8-11] | 70 | 9.5 / 11.3 | 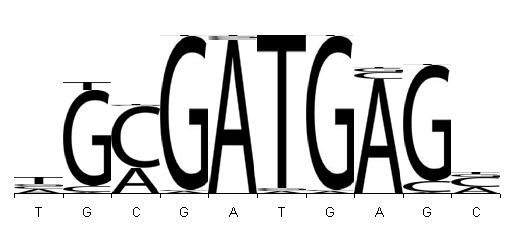  (<0.8 correlation coefficient)  (64/164 genes, 4.5) | rRNA transcription / Synthesis  (*for MDscan Motif Genes, P ≥ 4.4*) | ≥ 4.3 |
|  |  | PAC | [3] | 60 | 5.0 / 9.0 |  | Translation | 3.6 |
|  |  | SWI4 | [1-6] | 50 | 3.0 / 4.0 |  | tRNA transcription / Synthesis  (*for MDscan Motif Genes, P ≥ 2.9*) | 3.5 |
|  |  | RRPE | [3] | 40 | 12.2 / ≥32 |  | Nucleus | 2.3 |
| C_N2_ 3 | 121 | ABF1 | [1-5, 8-11] | 70 | 4.3 / 4.7 | 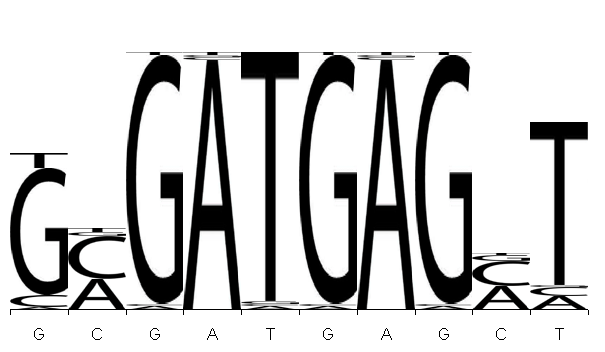  PAC^[7]^ (1.0)  (73/121 genes, 5.1) | rRNA transcription / Processing  (*for MDscan Motif Genes, P ≥ 5.3*) | ≥ 7.6 |
|  |  | PAC | [3] | 70 | 10.4 / 12.7 |  | Pyrimidine ribonucleotide metabolism | 3.7 |
|  |  | RRPE | [3] | 50 | 11.5 / 11.3 |  | Protein synthesis | 2.9 |
|  |  |  |  |  |  |  | Ribosome biogenesis | 2.6 |
| C_N2_ 4 | 265 | PAC | [3] | 60 | 10.1 / 15.0 | 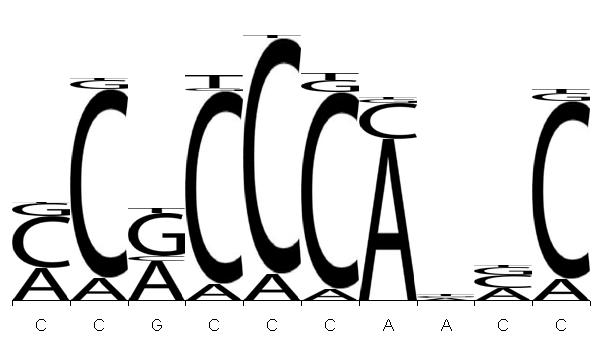  RAP1^[2]^ (0.83)  (106/266 genes, 3.9) | Ribosome biogenesis  *(for MDscan Motif Genes, P = 14 )* | 14.0 |
|  |  | ABF1 | [1] | 30 | 6.7 / 11.1 |  | Protein synthesis  *(for MDscan Motif Genes, P = 14 )* | 14.0 |
|  |  | RRPE | [3] | 30 | 7.2 / 11.5 |  | Purine ribonucleotide metabolism | 10.8 |
|  |  |  |  |  |  |  | rRNA processing / Transcription | ≥ 7.7 |
| C_N2_ 5 | 226 | HAP1 | [2, 3, 5, 8-10, 12-14] | 30 | 9.1 / 7.4 | 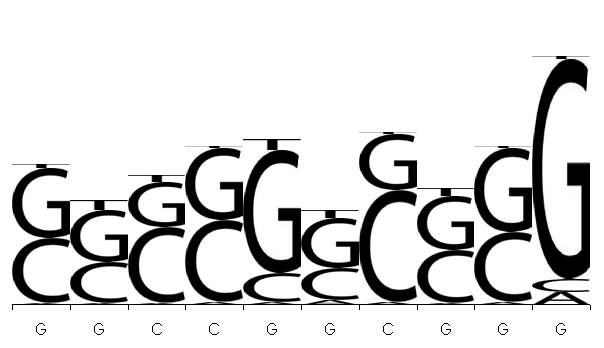SUT1^[2]^ (0.85)  (86/226 genes, 4.5) | Energy / Mitochondrion / Respiration  *(for MDscan Motif Genes, P ≥ 7.1 )* | 14.0 |
|  |  |  |  |  |  |  | Ionic homeostasis  *(for MDscan Motif Genes, P = 4.6 )* | 7.0 |
|  |  |  |  |  |  |  | Mitochondrial transport  *(for MDscan Motif Genes, P = 3.9 )* | 6.9 |
|  |  |  |  |  |  |  | Transport facilitation  *(for MDscan Motif Genes, P = 4.4 )* | 6.2 |
| C_N2_ 6 | 154 | 3’-PUF3 | [12, 15] | 60 | 22.7 / 24.2 | 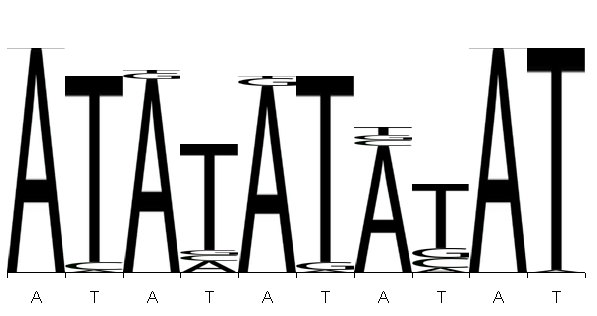 (<0.8 correlation coefficient)  (55/154 genes, 4.0) | Mito./ Ribo. biogen. / Protein synth.  *(for MDscan Motif Genes, P ≥ 5.5 )* | 14.0 |
|  |  | 3’-Motif6 | [3] | 20 | 11.7 / ≥32 |  | Energy / Respiration | ≥ 3.6 |
|  |  | 3’-CYTGTAAATA | [16] | 10 | 9.3 / 8.4 |  | Tricarboxylic-acid pathway | 3.5 |
|  |  |  |  |  |  |  | Assembly of protein complexes | 2.7 |
| C_N2_ 7 | 94 | UME6 | [2, 12, 17, 18] | 10 | 2.1 / 2.2 | 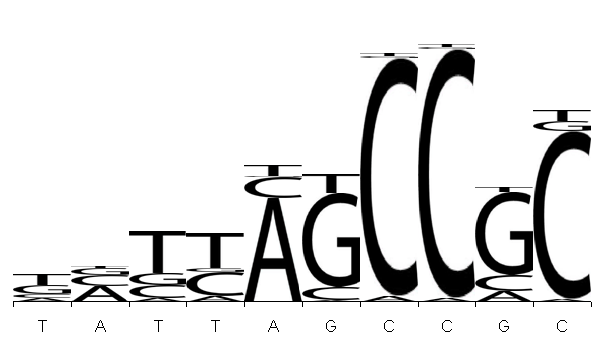UME6^[19]^ (0.93)  (55/94 genes, 3.4) | Amino acid metabolism | 2.5 |
|  |  |  |  |  |  |  | Lipid, fatty-acid and isoprenoid utilization | 2.2 |
|  |  |  |  |  |  |  | Protein folding and stabilization | 2.8 |
|  |  |  |  |  |  |  |  |  |

*Continued on following page*

*TABLE 1 − Continued*

| **Cluster** | **Gene**  **no*.*** | **TFM** | **Reference(s)** | **Share (%)** | ***P*-value*^b^***  **(set/genome)** | **MDscan motif logo (corr.)**  **(freq. & score)** | **MIPS functional category enrichment** | ***P*-value*^b^***  **(genomic)** |
| --- | --- | --- | --- | --- | --- | --- | --- | --- |
| C_N2_ 8 | 147 | STRE*^d^* | [9] | 70 | 4.5 / 7.1 | 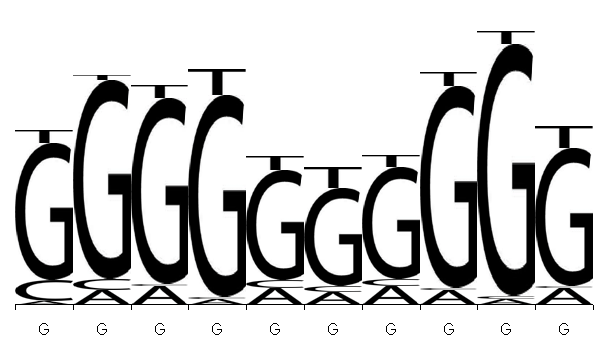STRE^[7]^ (0.91)  (34/147 genes, 4.3) | C-cmpd. & carbo. metab. / Transport  *(for MDscan Motif Genes, P ≥ 3.3 )* | ≥ 5.1 |
|  |  | MSN2/4 | [2] | 60 | 6.2 / 9.4 |  | Proteolytic degradation  *(for MDscan Motif Genes, P = 2.7 )* | 6.1 |
|  |  | MIG1 | [3] | 20 | 2.1 / 3.0 |  | Lysosomal and vacuolar degradation | 4.2 |
|  |  |  |  |  |  |  | Metabolism of energy reserves | 3.7 |
| C_N2_ 9 | 132 | STRE*^d^* | [3, 8, 9, 20] | 80 | 10.0 / 9.2 | 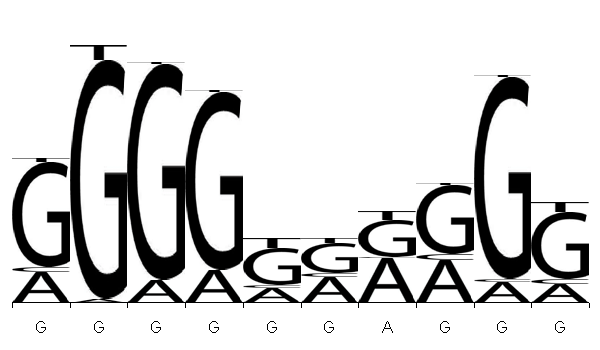STRE^[7]^ (0.84)  (76/132 genes, 4.0) | C-cmpd. & carbo. metab. / Utilization  *(for MDscan Motif Genes, P ≥ 7.1 )* | ≥ 6.4 |
|  |  | MSN2/4 | [21] | 60 | 10.5 / 10.7 |  | Metabolism of energy reserves  *(for MDscan Motif Genes, P = 9.5 )* | 8.6 |
|  |  |  |  |  |  |  | C-cmpd, carbo. transport(ers)  *(for MDscan Motif Genes, P ≥ 2.2 )* | ≥ 2.6 |
|  |  |  |  |  |  |  | Cellular import | 2.3 |
| C_N2_ 10 | 78 | CBF1/PHO4 | [3, 22] | 20 | 2.5 / 3.7 | 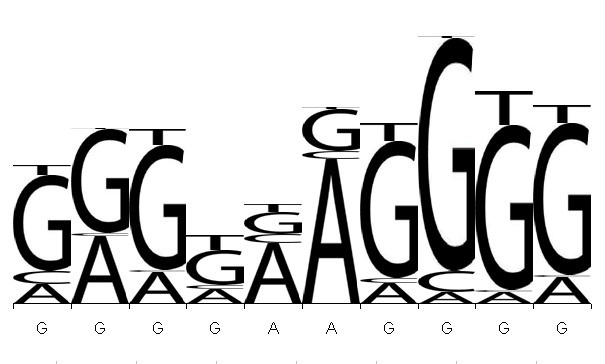  STRE^[7]^ (0.84)  (45/78 genes, 3.3) | C-cmpd. & carbo. metab. / Utilization  *(for MDscan Motif Genes, P ≥ 2.2 )* | ≥ 3.3 |
|  |  | MET31 | [1] | 10 | 3.0 / 4.5 |  | Metabolism | 3.1 |
|  |  | MET4 | [1] | 10 | 2.4 / 3.4 |  | Regulation of nitrogen & sulfur utilization / Metabolism | ≥ 2.3 |
|  |  |  |  |  |  |  |  |  |
| C_N2_ 11 | 67 | ROX1 | [8, 9] | 30 | 3.4 / 2.7 | 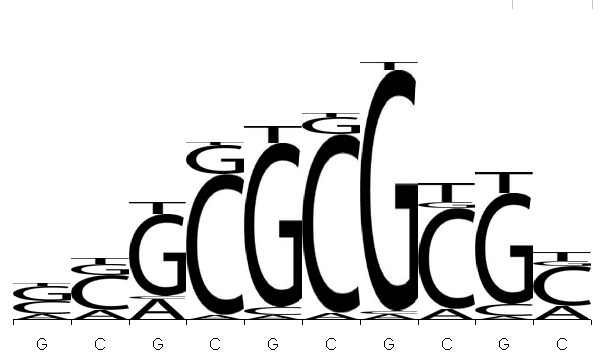  (<0.8 correlation coefficient)  (16/68 genes, 3.6) | Glycolysis and gluconeogenesis | 7.2 |
|  |  | YCTATTGTT | [3] | 20 | 3.0 / 4.0 |  | Amino acid biosynthesis | 4.4 |
|  |  | MET31 | [1] | 10 | 2.1 / 2.5 |  | Regulation of carbo. utilization / Metabolism | ≥ 2.8 |
|  |  | MET4 | [1] | 10 | 3.5 / 3.0 |  | Nitrogen and sulfur utilization | 3.1 |
| C_N2_ 12 | 98 | UPC2 | [23, 24] | 40 | 2.2 / NS | 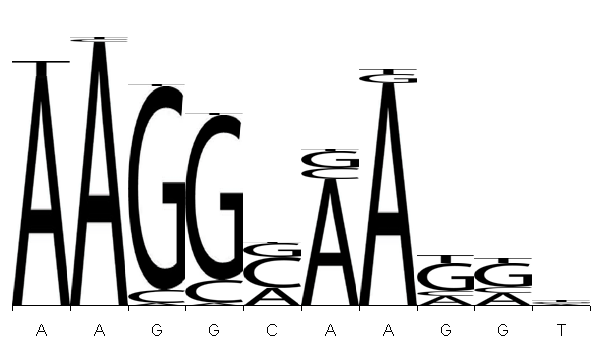 (<0.8 correlation coefficient)  (69/98 genes, 3.4) | C-cmpd. & carbo. metab. / Utilization  *(for MDscan Motif Genes, P ≥ 2.8 )* | ≥ 2.3 |
|  |  | ROX1 | [2] | 20 | 2.3 / 2.3 |  | Extracellular / secretion proteins | 3.6 |
|  |  |  |  |  |  |  | Biosynth. vitam., cofact. & prosth.  *(for MDscan Motif Genes, P = 2.6 )* | 3.4 |
|  |  |  |  |  |  |  | Glycolysis and gluconeogenesis | 2.7 |
| C_N2_ 13 | 125 | UPC2 | [23, 24] | 60 | 12.0 / 10.2 | 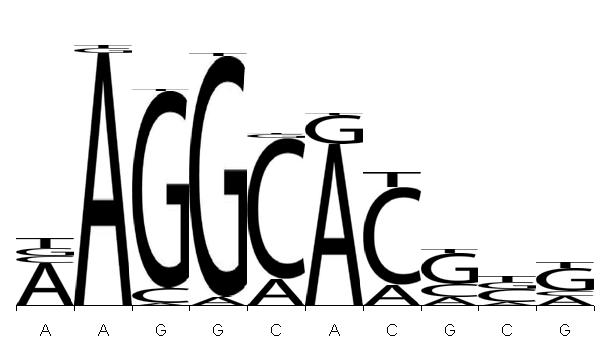  PHD1^[19]^ (0.83)  (91/125 genes, 4.0) | Stress response  *(for MDscan Motif Genes, P = 8.4 )* | 6.5 |
|  |  | ROX1 | [2] | 20 | 2.2 / 2.7 |  | Cell rescue, defense and virulence  *(for MDscan Motif Genes, P = 7.5 )* | 6.3 |
|  |  |  |  |  |  |  | Cell wall  *(for MDscan Motif Genes, P = 5.5 )* | 4.0 |
|  |  |  |  |  |  |  | Lipid, FA & isoprenoid biosynthesis / Metabolism | ≥ 2.5 |

*^a^* SOM clustering with 1D ring topology (*K* = 13).

*^b^* *p*-value (-log_10_(*p*)) for TFM enrichment relative to clustered genes (1754) / *p*-value (-log_10_(*p*)) for TFM enrichment relative to genome.

*^c^* *p*-value (-log_10_(*p*)) for MIPS functional category enrichment.

*^d^* STRE (AGGGG) has been listed as the binding site for Msn2, Msn4, Gis1 and/or Rph1 by different authors (citations above).

**TABLE 2.**  Selected list of enriched consensus sequence motifs (TFMs), MDscan sequence logos, and MIPS functional categories in clusters*^a^* of genes differentially expressed in response to antimycin A in air.

| **Cluster** | **Gene**  **no*.*** | **TFM** | **Reference(s)** | **Share (%)** | ***P*-value*^b^***  **(set/genome)** | **MDscan motif logo (corr.)**  **(freq. & score)** | **MIPS functional category enrichment** | ***P*-value*^c^***  **(genomic)** |
| --- | --- | --- | --- | --- | --- | --- | --- | --- |
| C_A-O2_ 1 | 30 | MCB | [8, 9] | 60 | 3.7 / 4.2 | 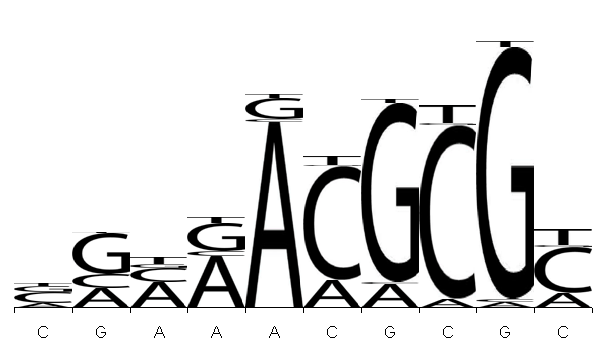  MBP1^[19]^ (0.94), SWI6^[19]^ (0.93)  (29/30 genes, 3.1) | Amino acid biosynthesis / Metabolism | 2.6 |
|  |  | MBP1 | [3, 5] | 30 | 4.4 / 4.1 |  | Metabolism | 2.6 |
|  |  | SWI4 | [1] | 30 | 4.0 / 3.5 |  | DNA synthesis and replication  *(for MDscan Motif Genes, P = 2.4)* | 2.0 |
|  |  | GCN4 | [4] | 20 | 3.5 / 3.1 |  |  |  |
| C_A-O2_ 2 | 121 | ABF1 | [1-5, 8-11] | 70 | 3.5 / 5.2 | 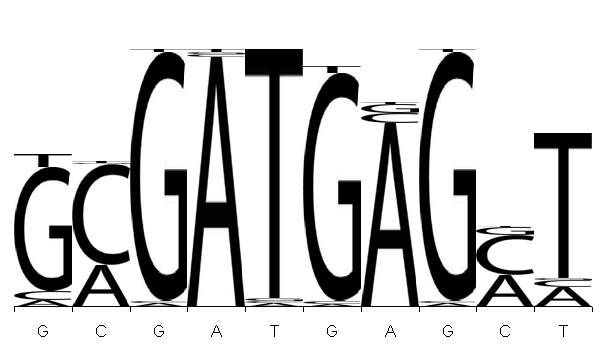PAC^[7]^ (1.0)  (56/121 genes, 4.8) | Nucleus  *(for MDscan Motif Genes, P = 3.8)* | 6.7 |
|  |  | PAC | [3] | 60 | 3.0 / 9.0 |  | rRNA transcription / Synthesis  *(for MDscan Motif Genes, P ≥ 2.4)* | ≥4.1 |
|  |  | MCB | [9] | 40 | 4.7 / 4.5 |  | DNA synthesis & replication / Process. | ≥3.5 |
|  |  | RRPE | [3] | 30 | 3.3 / 8.0 |  | Cell cycle and DNA processing | 4.3 |
| C_A-O2_ 3 | 310 | PAC | [3] | 70 | 14.2 / 23.1 | 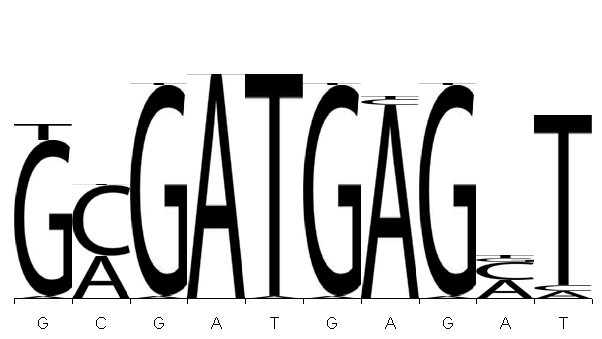PAC^[7]^ (1.0)  (130/310 genes, 6.0) | rRNA transcription / Processing  *(for MDscan Motif Genes, P = 14.0)* | ≥13.2 |
|  |  | ABF1 | [1-5, 8-11] | 60 | 5.0 / 7.3 |  | Protein synthesis / Ribosome biogenesis | ≥10.3 |
|  |  | RRPE | [3] | 40 | 12.4 / ≥32 |  | tRNA transcript. / Modification / Synth.  *(for MDscan Motif Genes, P = 2.2)* | ≥2.6 |
|  |  |  |  |  |  |  | Purine / Pyrimidine ribonucleotide metabolism | ≥4.2 |
| C_A-O2_ 4 | 255 | STRE*^d^* | [3, 8, 9, 20] | 80 | 19.0 / 28.5 | 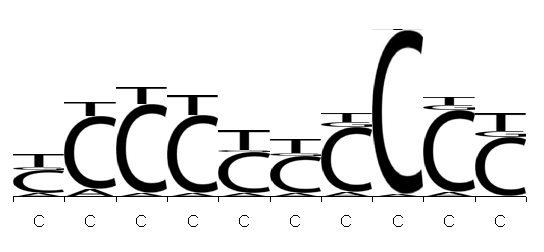STRE^[7]^ (0.93)  (85/255 genes, 4.7) | Metabolism of energy reserves  *(for MDscan Motif Genes, P = 2.1)* | 10.2 |
|  |  | MSN2/4 | [2] | 70 | 26.5 / 34.5 |  | C-cmpd. & carbo. metab. / Util. / Trans.  *(for MDscan Motif Genes, P = 2.0)* | ≥4.1 |
|  |  | MIG1 | [3] | 30 | 2.7 / 4.7 |  | Energy  *(for MDscan Motif Genes, P = 2.7)* | 7.1 |
|  |  |  |  |  |  |  | Stress response | 3.6 |
| C_A-O2_ 5 | 101 | STRE*^d^* | [3, 8, 9, 20] | 70 | 3.0 / 7.5 | 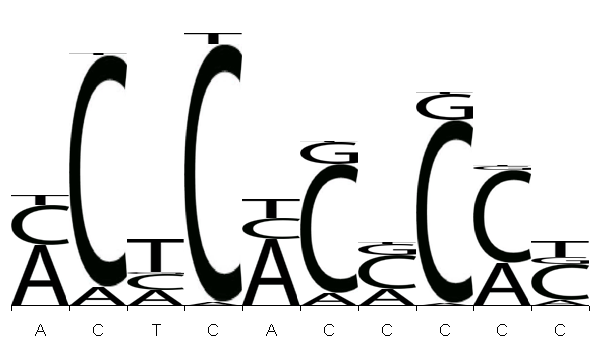STRE^[7]^ (0.76)  (80/101 genes, 3.8) | C-compd. & carbo. metab. / Utilization  *(for MDscan Motif Genes, P ≥ 6.2)* | ≥9.8 |
|  |  | ADR1/MIG1 | [10] | 20 | 3.0 / 2.3 |  | Energy  *(for MDscan Motif Genes, P = 4.8)* | 6.4 |
|  |  |  |  |  |  |  | Amino acid metabolism  *(for MDscan Motif Genes, P = 3.3)* | 4.0 |
|  |  |  |  |  |  |  | Stress response  *(for MDscan Motif Genes, P = 3.0)* | 4.0 |
| C_A-O2_ 6 | 84 | GCN4 | [2] | 20 | 2.5 / 3.0 | 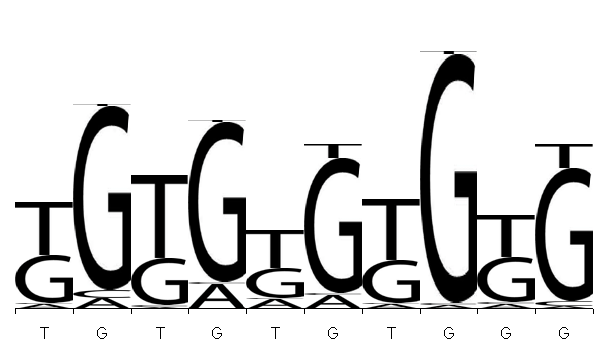  (<0.8 correlation coefficient)  (39/84 genes, 4.1) | Energy  *(for MDscan Motif Genes, P = 2.3)* | 6.0 |
|  |  | MIG1 | [3] | 20 | 3.0 / 4.3 |  | Glycolysis and gluconeogenesis | 5.2 |
|  |  |  |  |  |  |  | C-compd. & carbo. metab. / Utilization  *(for MDscan Motif Genes, P ≥ 2.3)* | ≥4.4 |
|  |  |  |  |  |  |  | Amino acid metabolism / Degradation  *(for MDscan Motif Genes, P ≥ 2.8)* | ≥2.9 |

*^a^* SOM clustering with 1D ring topology (*K* = 6).

*^b^* *p*-value (-log_10_(*p*)) for TFM enrichment relative to clustered genes (901) / *p*-value (-log_10_(*p*)) for TFM enrichment relative to genome.

*^c^* *p*-value (-log_10_(*p*)) for MIPS functional category enrichment.

*^d^* STRE (AGGGG) has been listed as the binding site for Msn2, Msn4, Gis1 and/or Rph1 by different authors (citations above).

**TABLE 3.** Selected list of enriched consensus sequence motifs (TFMs), MDscan sequence logos, and MIPS functionalcategories in clusters*^e^* of genes differentially expressed in response to anaerobiosis in the presence of antimycin A.

| **Cluster** | **Gene**  **no*.*** | **TFM** | **Reference(s)** | **Share (%)** | ***P*-value*^b^***  **(set/genome)** | **MDscan motif logo (corr.)**  **(freq. & score)** | **MIPS functional category enrichment** | ***P*-value*^c^***  **(genomic)** |
| --- | --- | --- | --- | --- | --- | --- | --- | --- |
| C_A-N2_ 1 | 22 | GCN4 | [4] | 30 | 2.5 / 2.7 | 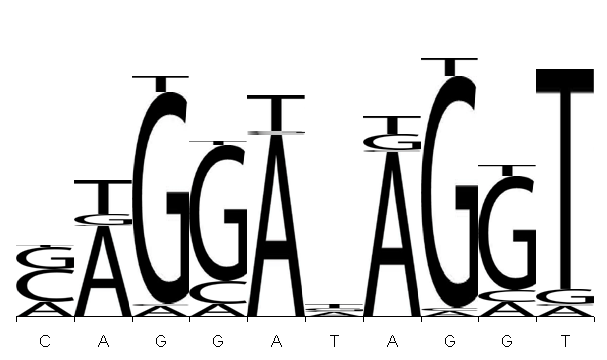  (<0.8 correlation coefficient)  (22/22 genes, 3.0) |  |  |
|  |  | M429*^e^*  (RRPE) | [25] | 20 | 2.7 / 2.5 |  |  |  |
|  |  |  |  |  |  |  |  |  |
|  |  |  |  |  |  |  |  |  |
| C_A-N2_ 2 | 41 | PUT3 | [1-5, 8, 9] | 40 | 3.5 / 3.5 | 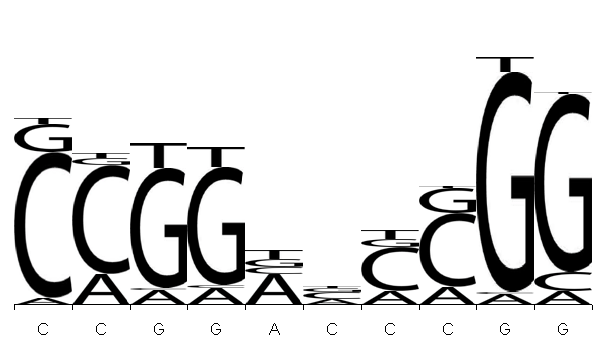  (<0.8 correlation coefficient)  (34/41 genes, 3.3) | Pyrimidine ribonucleotide metabolism | 4.5 |
|  |  | HAP1 | [12, 13] | 20 | 2.1 / 2.7 |  | Lipid, FA & isopren. Synth. / Metab.  *(for MDscan Motif Genes, P ≥ 2.2)* | ≥2.6 |
|  |  |  |  |  |  |  | Nitrogen and sulfur util. / Metab.  *(for MDscan Motif Genes, P = 2.2)* | ≥2.0 |
|  |  |  |  |  |  |  | Amino acid biosynthesis  *(for MDscan Motif Genes, P = 2.2)* | 2.2 |
| C_A-N2_ 3 | 53 | PUT3 | [10] | 40 | 2.2 / 3.4 | 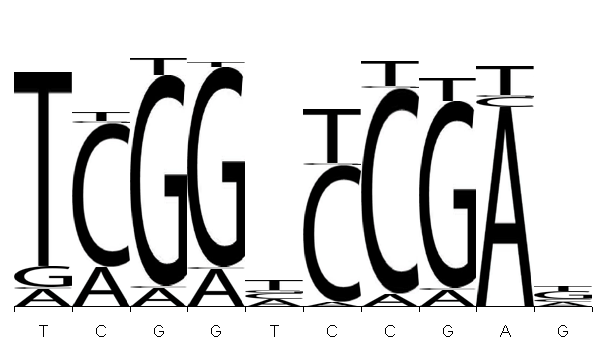  (<0.8 correlation coefficient)  (24/53 genes, 3.2) | Respiration  *(for MDscan Motif Genes, P = 2.5)* | 10.0 |
|  |  | 3’-CTH2 | [26] | 30 | 3.0 / 3.5 |  | Mitochondrion | 8.5 |
|  |  |  |  |  |  |  | Energy  *(for MDscan Motif Genes, P = 2.1)* | 7.0 |
| C_A-N2_ 4 | 89 | 3’-PUF3 | [12, 15] | 50 | 10.0 / 9.5 | 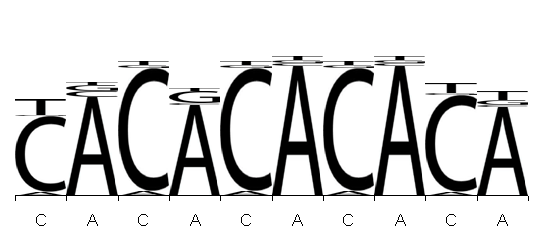MET31^[19]^ (0.81)  (8/89 genes, 4.1) | Mitochondrion  *(for MDscan Motif Genes, P = 2.3)* | 14.0 |
|  |  | PUT3 | [10] | 40 | 3.3 / 5.4 |  | Respiration | 7.5 |
|  |  | 3’-Motif6 | [3] | 20 | 6.0 / 5.2 |  | Energy |  |
|  |  |  |  |  |  |  | Protein synthesis | 3.9 |
| C_A-N2_ 5 | 39 | GGAAARRGRW | [25] | 30 | 2.4 / 2.0 | 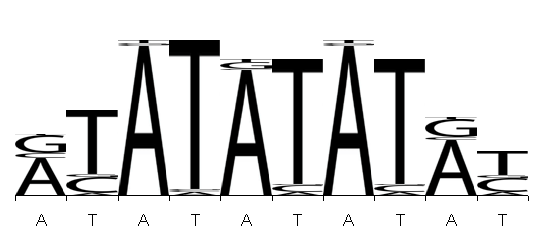AT repeat^[7]^ (0.93)  (16/39 genes, 3.1) | Amino acid metabolism / Biosynthesis *(for MDscan Motif Genes, P ≥ 3.3)* | ≥2.2 |
|  |  | M776 *^e^*  (RRPE) | [25] | 20 | 2.0 / 2.0 |  | Oxidation of fatty acids | 3.1 |
|  |  |  |  |  |  |  | Mitochondrion | 2.8 |
|  |  |  |  |  |  |  | Nitrogen and sulfur metabolism | 2.0 |
| C_A-N2_ 6 | 95 | SWI6 | [2] | 20 | 3.5 / 3.2 | 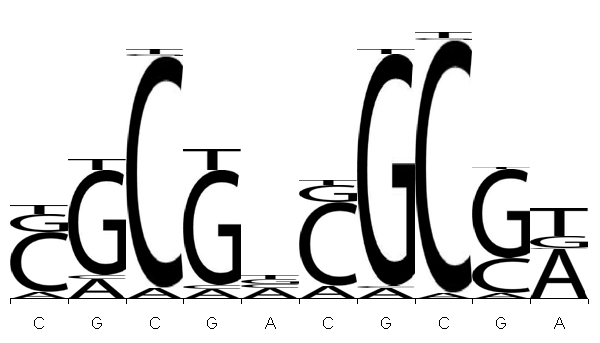  (<0.8 correlation coefficient)  (31/95 genes, 3.7) | Metabolism | 7.3 |
|  |  |  |  |  |  |  | C-compd. & carbo. metab. / Utilization *(for MDscan Motif Genes, P = 2.5)* | ≥5.0 |
|  |  |  |  |  |  |  | Extracellular / Secretion proteins  *(for MDscan Motif Genes, P = 2.6)* | 2.5 |
| C_A-N2_ 7 | 116 | ROX1 | [8, 9] | 30 | 7.0 / 4.2 | 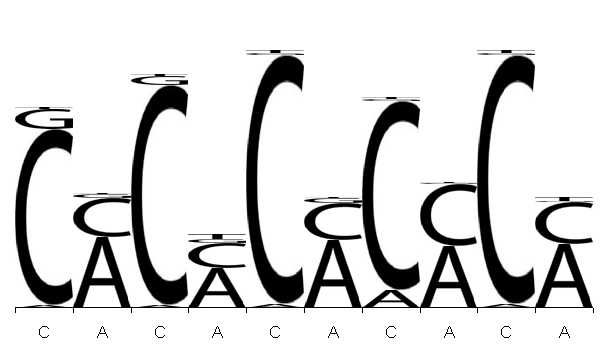STRE^[7]^ (0.80)  (35/116 genes, 4.3) | Metabolism | 7.7 |
|  |  |  |  |  |  |  | Metabolism of energy reserves  *(for MDscan Motif Genes, P = 3.5)* | 6.5 |
|  |  |  |  |  |  |  | C-compd. & carbo. metab. / Utilization | ≥5.2 |
|  |  |  |  |  |  |  | Energy | 4.2 |

*Continued on following page*

TABLE 3 − *Continued*

| **Cluster** | **Gene**  **no*.*** | **TFM** | **Reference(s)** | **Share (%)** | | ***P*-value*^b^***  **(set/genome)** | **MDscan motif logo (corr.)**  **(freq. & score)** | **MIPS functional category enrichment** | | ***P*-value*^c^***  **(genomic)** |
| --- | --- | --- | --- | --- | --- | --- | --- | --- | --- | --- |
| C_A-N2_ 8 | 53 | MSN2/4 | [2] | 60 | 2.5 / 2.4 | | 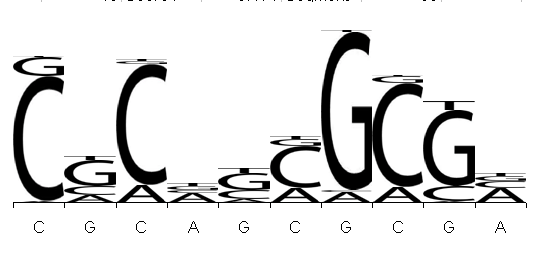SUT1^[19]^ (0.80)  (26/53 genes, 3.2) | Stress response  *(for MDscan Motif Genes, P = 4.0)* | 4.9 | |
|  |  |  |  |  |  | |  | Cell rescue, defense and virulence  *(for MDscan Motif Genes, P = 2.9)* | 4.8 | |
|  |  |  |  |  |  | |  | C-compd. & carbo. metab. / Utilization | ≥3.5 | |
| C_A-N2_ 9 | 101 | UPC2 | [23, 24] | 60 | 8.0 / 11.7 | | 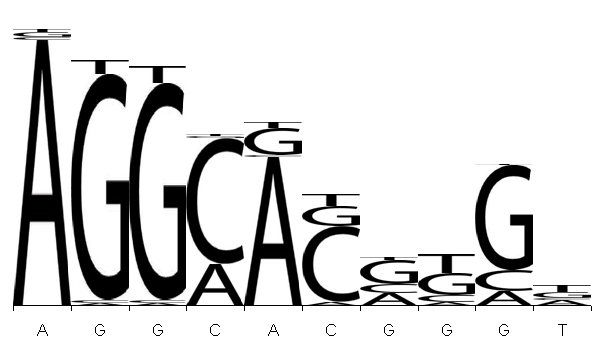PHD1^[19]^ (0.86),  MOT3^[2]^ (0.81)  (64/101 genes, 3.6) | Stress response  *(for MDscan Motif Genes, P = 7.3)* | 14.0 | |
|  |  | AFT2 | [2, 10] | 50 | 3.0 / 4.1 | |  | Cell rescue, defense and virulence  *(for MDscan Motif Genes, P = 5.3)* | 10.4 | |
|  |  |  |  |  |  | |  | Cell wall  *(for MDscan Motif Genes, P = 3.7)* | 4.6 | |
|  |  |  |  |  |  | |  | Extracellular / Secretion proteins  *(for MDscan Motif Genes, P = 2.0)* | 3.6 | |
| C_A-N2_ 10 | 60 | RPH1 | [2, 4, 10, 12] | 30 | 2.1 / 2.7 | | 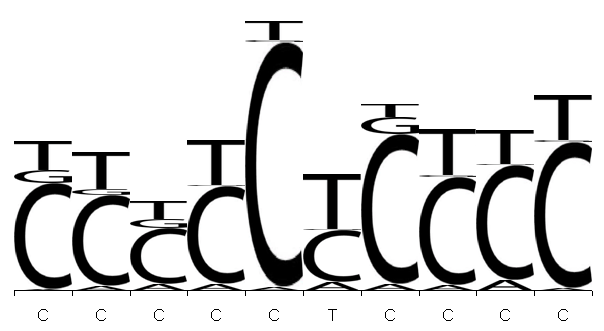  STRE^[7]^ (0.88)  (33/60 genes, 3.4) | Amino acid biosynthesis / Metabolism | ≥4.7 | |
|  |  | YAP5 | [27] | 20 | 2.2 / NS | |  | Metabolism | 5.3 | |
|  |  | GCN4 | [1] | 10 | 2.7 / 3.5 | |  | Lipid, FA and isoprenoid biosynthesis / Metabolism | ≥2.4 | |
|  |  |  |  |  |  | |  | Detoxification with cytochrome p450  *(for MDscan Motif Genes, P = 4.0)* | 3.3 | |

*^a^* SOM clustering with 1D ring topology (*K* = 10).

*^b^* *p*-value (-log_10_(*p*)) for TFM enrichment relative to clustered genes (669) / *p*-value (-log_10_(*p*)) for TFM enrichment relative to genome.

*^c^* *p*-value (-log_10_(*p*)) for MIPS functional category enrichment.

*^d^* STRE (AGGGG) has been listed as the binding site for Msn2, Msn4, Gis1 and/or Rph1 by different authors (citations above).

*^e^* See supplementary Table S4 for explanation of numbered motifs: F = functional motif; C = coherent motif from reference [28].

References

1. Grishin AV, Rothenberg M, Downs MA, Blumer KJ: **Mot3, a Zn finger transcription factor that modulates gene expression and attenuates mating pheromone signaling in *Saccharomyces cerevisiae***. *Genetics* 1998, **149:**879-892.

2. Harbison CT, Gordon DB, Lee TI, Rinaldi NJ, Macisaac KD, Danford TW, Hannett NM, Tagne JB, Reynolds DB, Yoo J, Jennings EG, Zeitlinger J, Pokholok DK, Kellis M, Rolfe PA, Takusagawa KT, Lander ES, Gifford DK, Fraenkel E, Young RA: **Transcriptional regulatory code of a eukaryotic genome**. *Nature* 2004, **431:**99-104.

3. Kellis M, Patterson N, Endrizzi M, Birren B, Lander ES: **Sequencing and comparison of yeast species to identify genes and regulatory elements**. *Nature* 2003, **423:**241-254.

4. Lee TI, Rinaldi NJ, Robert F, Odom DT, Bar-Joseph Z, Gerber GK, Hannett NM, Harbison CT, Thompson CM, Simon I, Zeitlinger J, Jennings EG, Murray HL, Gordon DB, Ren B, Wyrick JJ, Tagne JB, Volkert TL, Fraenkel E, Gifford DK, Young RA: **Transcriptional regulatory networks in *Saccharomyces cerevisiae***. *Science* 2002, **298:**799-804.

5. Svetlov VV, Cooper TG: **Review: compilation and characteristics of dedicated transcription factors in *Saccharomyces cerevisiae***. *Yeast* 1995, **11:**1439-1484.

6. Taba MR, Muroff I, Lydall D, Tebb G, Nasmyth K: **Changes in a SWI4,6-DNA-binding complex occur at the time of HO gene activation in yeast**. *Genes Dev* 1991, **5:**2000-2013.

7. Hughes JD, Estep PW, Tavazoie S, Church GM: **Computational identification of *cis*-regulatory elements associated with groups of functionally related genes in *Saccharomyces cerevisiae***. *J Mol Biol* 2000, **296:**1205-1214.

8. Zhu J, Zhang MQ: **SCPD: a promoter database of the yeast *Saccharomyces cerevisiae***. *Bioinformatics* 1999, **15:**607-611.

9. Xing B, van der Laan MJ: **A statistical method for constructing transcriptional regulatory networks using gene expression and sequence data**. *J Comput Biol* 2005, **12:**229-246.

10. Wang T, Stormo GD: **Identifying the conserved network of cis-regulatory sites of a eukaryotic genome**. *Proc Natl Acad Sci U S A* 2005.

11. de Winde JH, Grivell LA: **Global regulation of mitochondrial biogenesis in *Saccharomyces cerevisiae*: ABF1 and CPF1 play opposite roles in regulating expression of the *QCR8* gene, which encodes subunit VIII of the mitochondrial ubiquinol-cytochrome *c* oxidoreductase**. *Mol Cell Biol* 1992, **12:**2872-2883.

12. Gasch AP, Moses AM, Chiang DY, Fraser HB, Berardini M, Eisen MB: **Conservation and evolution of cis-regulatory systems in ascomycete fungi**. *PLoS Biol* 2004, **2:**e398.

13. King DA, Zhang L, Guarente L, Marmorstein R: **Structure of a HAP1-DNA complex reveals dramatically asymmetric DNA binding by a homodimeric protein**. *Nat Struct Biol* 1999, **6:**64-71.

14. Vuidepot AL, Bontems F, Gervais M, Guiard B, Shechter E, Lallemand JY: **NMR analysis of CYP1(HAP1) DNA binding domain-CYC1 upstream activation sequence interactions: recognition of a CGG trinucleotide and of an additional thymine 5 bp downstream by the zinc cluster and the N- terminal extremity of the protein**. *Nucleic Acids Res* 1997, **25:**3042-3050.

15. Gerber AP, Herschlag D, Brown PO: **Extensive association of functionally and cytotopically related mRNAs with Puf family RNA-binding proteins in yeast**. *PLoS Biol* 2004, **2:**E79.

16. Jacobs Anderson JS, Parker R: **Computational identification of cis-acting elements affecting post-transcriptional control of gene expression in *Saccharomyces cerevisiae***. *Nucleic Acids Res* 2000, **28:**1604-1617.

17. Bernstein BE, Tong JK, Schreiber SL: **Genomewide studies of histone deacetylase function in yeast**. *Proc Natl Acad Sci U S A* 2000, **97:**13708-13713.

18. Wang Y, Pierce M, Schneper L, Guldal CG, Zhang X, Tavazoie S, Broach JR: **Ras and gpa2 mediate one branch of a redundant glucose signaling pathway in yeast**. *PLoS Biol* 2004, **2:**E128.

19. Macisaac KD, Wang T, Gordon DB, Gifford DK, Stormo GD, Fraenkel E: **An improved map of conserved regulatory sites for Saccharomyces cerevisiae**. *BMC Bioinformatics* 2006, **7:**113.

20. Jang YK, Wang L, Sancar GB: **RPH1 and GIS1 are damage-responsive repressors of PHR1**. *Mol Cell Biol* 1999, **19:**7630-7638.

21. Martinez-Pastor MT, Marchler G, Schüller C, Marchler-Bauer A, Ruis H, Estruch F: **The *Saccharomyces cerevisiae* zinc finger proteins Msn2p and Msn4p are required for transcriptional induction through the stress response element (STRE)**. *EMBO J* 1996, **15:**2227-2235.

22. Kent NA, Eibert SM, Mellor J: **Cbf1p is required for chromatin remodeling at promoter-proximal CACGTG motifs in yeast**. *J Biol Chem* 2004, **279:**27116-27123.

23. Cohen BD, Sertil O, Abramova NE, Davies KJ, Lowry CV: **Induction and repression of *DAN1* and the family of anaerobic mannoprotein genes in *Saccharomyces cerevisiae* occurs through a complex array of regulatory sites**. *Nucleic Acids Res* 2001, **29:**799-808.

24. Vik A, Rine J: **Upc2p and Ecm22p, dual regulators of sterol biosynthesis in *Saccharomyces cerevisiae***. *Mol Cell Biol* 2001, **21:**6395-6405.

25. Pritsker M, Liu YC, Beer MA, Tavazoie S: **Whole-genome discovery of transcription factor binding sites by network-level conservation**. *Genome Res* 2004, **14:**99-108.

26. Puig S, Askeland E, Thiele DJ: **Coordinated remodeling of cellular metabolism during iron deficiency through targeted mRNA degradation**. *Cell* 2005, **120:**99-110.

27. Horak CE, Luscombe NM, Qian J, Bertone P, Piccirrillo S, Gerstein M, Snyder M: **Complex transcriptional circuitry at the G1/S transition in *Saccharomyces cerevisiae***. *Genes Dev* 2002, **16:**3017-3033.

28. Cliften P, Sudarsanam P, Desikan A, Fulton L, Fulton B, Majors J, Waterston R, Cohen BA, Johnston M: **Finding Functional Features in *Saccharomyces* Genomes by Phylogenetic Footprinting**. *Science* 2003, **301:**71-76.
