# Supplementary material for: Comparison of the transcriptomic "stress response" evoked by antimycin A and oxygen deprivation in saccharomyces cerevisiae
Source: BMC Genomics. 2008 Dec 23;9:627. doi: 10.1186/1471-2164-9-627 (PMC2637875; doi:10.1186/1471-2164-9-627)

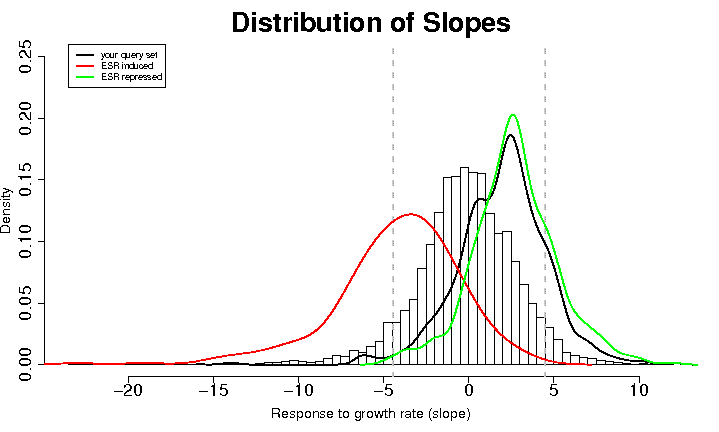
Additional file 7 – Figure 1

C_N2_1 – 4: Transiently Repressed Genes

Additional file 7 – Figure 2

C_N2_5 – 6: Chronically Repressed Genes


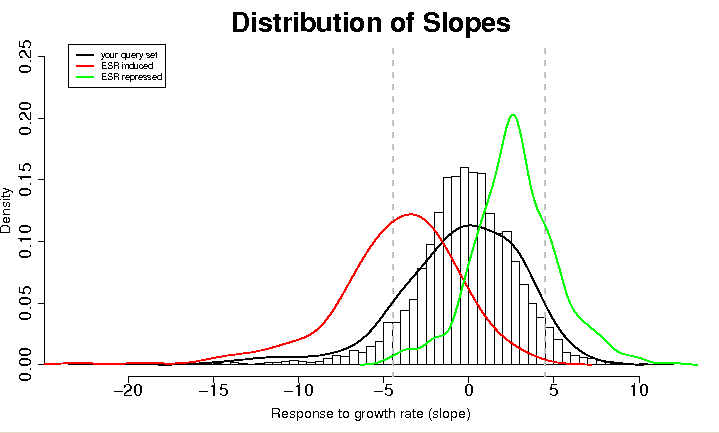


Additional file 7 – Figure 3

C_N2_7 – 9: Transiently Induced Genes


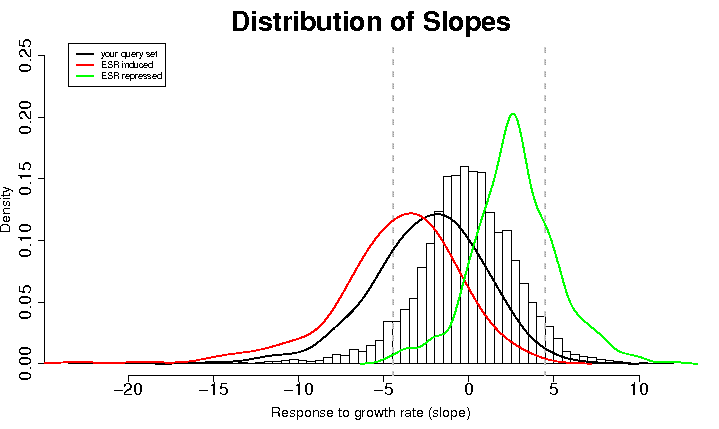
Additional file 7 – Figure 4

C_N2_10 – 13: Chronically Induced Genes


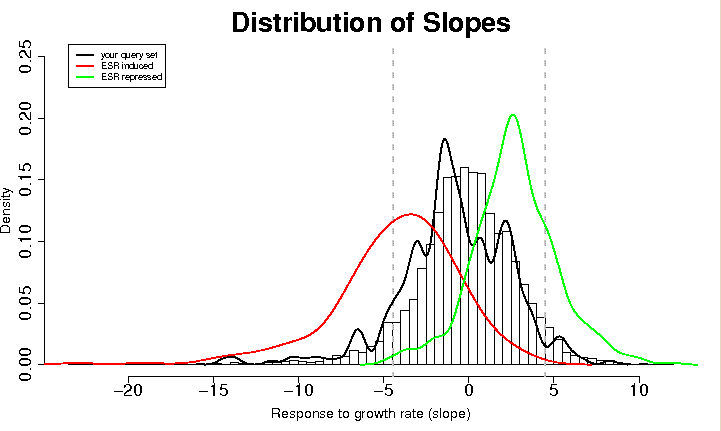

Supplement: Additional file 7 — Distribution of regression slopes for genes that were acutely repressed (Figure 1), chronically repressed (Figure 2), acutely induced (Figure 3), and chronically induced (Figure 4) in response to anaerobiosis. The graphs show the distribution of growth-rate slopes for specific gene clusters from the anaerobic dataset (black) in comparison to ESR-induced (red) and ESR-repressed genes (green). The graphs were generated using the online utility developed by Brauer et al. 2008 [9]. [file 1471-2164-9-627-S7.doc]
